# Magnetocrystalline Anisotropic Platinum–Palladium–Iron Ternary Intermetallic Alloy for Enhanced Fuel Cell Electrocatalysis

**DOI:** 10.1002/adma.202510314

**Published:** 2025-07-28

**Authors:** Muhammad Irfansyah Maulana, Jungho Kim, Ha‐Young Lee, Caleb Gyan‐Barimah, Yi Wei, Jeong‐Hoon Yu, Jong Hun Sung, Bo Yu, Kug‐Seung Lee, Seoin Back, Jong‐Sung Yu

**Affiliations:** ^1^ Department of Energy Science and Engineering Daegu Gyeongbuk Institute of Science and Technology (DGIST) Daegu 42988 Republic of Korea; ^2^ Department of Chemical and Biomolecular Engineering Sogang University Seoul 04107 Republic of Korea; ^3^ UE Science Inc. 66 Inan‐gil Gaejin‐myeon Goryeong‐gun Gyeongsangbuk‐do 40150 Republic of Korea; ^4^ Pohang Accelerator Laboratory (PAL) Pohang University of Science and Technology (POSTECH) Pohang 37673 Republic of Korea; ^5^ KU‐KIST Graduate School of Converging Science and Technology Korea University Seoul 02841 Republic of Korea; ^6^ Department of Integrative Energy Engineering Korea University Seoul 02841 Republic of Korea

**Keywords:** electrocatalysis, intermetallic catalyst, oxygen reduction reaction, polymer electrolyte membrane fuel cells, ternary alloy

## Abstract

Ordered Pt‐based intermetallic alloys have emerged as promising candidates for oxygen reduction reaction (ORR) electrocatalysts in comparison to their disordered counterparts. Here, novel ferromagnetic PtPdFe ternary intermetallic alloys with structurally ordered tetragonal L1_0_ and cubic L1_2_ phases are presented, featuring distinctive characteristics in crystal structures and atomic alignments. Insights into the fundamental understanding of the Pt‐based ternary intermetallic catalysts are provided, unveiling magnetocrystalline anisotropy as a structure‐intrinsic descriptor for ORR catalysis. Electrochemical half‐ and single‐cell assessments reveal that the L1_0_‐PtPdFe intermetallic catalysts exhibit superior ORR performance compared to their L1_2_‐type counterparts. Combined experimental and theoretical investigations indicate that the unique tetragonal structure of L1_0_‐PtPdFe, characterized by strong 5d–3d orbital interactions along the *c*‐axis direction, induces ferromagnetic ordering and leads to increased magnetocrystalline anisotropy energy, thereby accelerating the ORR process. The fuel cell fabricated by such a cathode catalyst retains its performance after prolonged degradation test, meeting the 2025 stability goals set by the US Department of Energy under H_2_–O_2_, H_2_–air, and H_2_–N_2_ conditions. These new conceptual findings establish a rational framework for designing high‐performance Pt‐based intermetallic electrocatalysts, where magnetic anisotropy arising from ferromagnetic ordering can be harnessed to tailor catalytic performance for next‐generation fuel cells.

## Graphical abstract

In this study, how crystallographic anisotropy governs the catalytic process is investigated by comparing the tetragonal L1_0_ and cubic L1_2_ ordered phases in a newly developed PtPdFe ternary intermetallic system. New insights into the fundamental understanding of the ordered Pt‐based ternary intermetallic alloys are introduced, unveiling magnetocrystalline anisotropy as a structure‐intrinsic descriptor for oxygen reduction electrocatalysis in fuel cells.

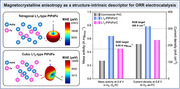

## 1 Introduction

The mass commercialization of polymer electrolyte membrane fuel cells (PEMFCs) hinges on the development of cost‐effective, high‐performance oxygen reduction reaction (ORR) electrocatalysts capable of withstanding harsh acidic environments.^[^ ^1^, ^2^ ^]^ The alloying of Pt with 3d transition metal (M) has been a subject of extensive exploration as a promising avenue to enhance ORR activity while reducing the consumption of Pt and the overall costs associated with the production of fuel cells.^[^ ^3^, ^4^ ^]^ Over the past decades, structurally ordered PtM intermetallic alloys have emerged as some of the most effective catalysts for PEMFC cathodes.^[^ ^5^, ^6^, ^7^ ^]^ Various PtM intermetallic nanoparticles (NPs), including PtFe,^[^ ^8^, ^9^, ^10^ ^]^ PtCo,^[^ ^11^, ^12^, ^13^, ^14^ ^]^ and PtNi,^[^ ^15^, ^16^, ^17^, ^18^ ^]^ have demonstrated superior ORR catalytic performance over their disordered counterparts, which is attributed to the stronger atomic interactions between Pt and M atoms.^[^ ^19^, ^20^, ^21^ ^]^ However, an excessive compressive strain in PtM intermetallics may alter the adsorption energy of reaction intermediates, potentially hindering reactant activation during the ORR process.^[^ ^22^, ^23^ ^]^ Additionally, prolonged fuel cell operation leads to M dissolution and particle disordering, resulting in substantial losses in intrinsic activity and durability.^[^ ^24^ ^–^ ^26^ ^]^

High‐temperature annealing of disordered A1‐type PtM alloys typically drives a disorder‐to‐order transition, forming either the L1_0_ face‐centered tetragonal (fct) or L1_2_ face‐centered cubic (fcc) phase under a well‐defined stoichiometry. These two phases possess fundamentally different crystallographic symmetries and atomic configurations, which in turn influence their electronic and surface chemistry. While strain engineering in PtM intermetallics has been widely investigated, the intrinsic impact of these diverse crystal structures on ORR activity remains insufficiently understood. A crucial yet often overlooked aspect in structurally ordered PtM alloys is the unique Pt–M 5d–3d hybridization, which modulates spin‐orbit coupling and contributes to the enhancement of magnetic anisotropy.^[^ ^27^, ^28^ ^]^ Given that this magnetic anisotropy is highly sensitive to factors such as lattice parameter, symmetry, atomic arrangement, and directional bonding, the L1_0_‐ and L1_2_‐PtM phases should display markedly different magnetic and catalytic behaviors. Previous reports have primarily focused on enhancing ORR via external magnetic fields by using extraneous magnetic supports or applying permanent magnets.^[^ ^29^, ^30^, ^31^, ^32^, ^33^ ^]^ Nevertheless, these approaches do not address the intrinsic role of magnetocrystalline effects in Pt intermetallic alloys–originating from structural ordering and internal magnetic interactions–in governing ORR catalysis.

In this study, we investigate the fundamental role of inherent magnetic anisotropy within structurally ordered Pt‐based intermetallics, establishing a direct link between crystallographic anisotropy and ORR efficiency. A structurally ordered PtPdFe ternary intermetallic alloy with L1_0_ and L1_2_ phases was synthesized via a solvothermal method followed by thermal heating treatment. An important consideration of the ternary design by integrating Pd into PtFe is to leverage the increased entropy effect to impede particle sintering. The incorporation of Fe into PtPd crystals triggers the ordering transformation of the alloy, imparting distinctive anisotropic properties. Our findings reveal that the L1_0_‐type PtPdFe alloy exhibits favorable magnetic characteristics for ORR compared to its L1_2_ counterpart, leading to enhanced cathodic reaction in PEMFC single‐cell test. Furthermore, the fuel cell fabricated by L1_0_‐PtPdFe cathode catalyst retained its performance after 30000 potential cycles, satisfying the 2025 durability targets set by the US Department of Energy (DOE) under H_2_–O_2_, H_2_–air, and H_2_–N_2_ operating conditions. This work provides new insights into the role of crystallographic anisotropy in ORR electrocatalysis and establishes a rational framework for designing high‐performance Pt‐based intermetallic catalysts for next‐generation PEMFCs.

## 2 Results and Discussion

### 2.1 Rational Design and Structural Characterization

Two types of magnetic intermetallic catalysts (MICs) were first prepared by reducing Pt, Pd, and Fe metal precursors via solvothermal reaction, generating disordered PtPdFe ternary alloys (Figure S1, Supporting Information). The Pt group metal (PGM)/Fe mole ratio was controlled at 1:1 and 3:1, denoted as A1‐PtPdFe‐11 and A1‐PtPdFe‐31, respectively. The X‐ray diffraction (XRD) peaks of A1‐PtPdFe‐11 closely matched those of PtFe standard, verifying successful alloy formation with a disordered A1 structure. On the other hand, a noticeable shift in peak positions toward lower 2θ angles was observed for A1‐PtPdFe‐31, approaching the Pt reference pattern. This shift is attributed to the increased PGM content of A1‐PtPdFe‐31, which alters the lattice parameters. To monitor the atomic composition and particle size evolution, ex situ transmission electron microscopy (TEM) and inductively coupled plasma‐optical emission spectroscopy (ICP‐OES) were employed. For A1‐PtPdFe‐11, nucleation of Pt/Pd/Fe was first detected at 200 °C, with an initial particle size of 2.52 nm (Figure S2a,b, Supporting Information), which gradually increased to 4–5 nm after 2 h of solvothermal reaction. At this stage, Pt and Pd were reduced more rapidly than Fe due to their higher redox potentials (Figure S2c, Supporting Information). The final PGM/Fe composition stabilized at 51.1/48.9 after 4 h at 200 °C (Figure S2d, Supporting Information), closely matching the initial 1:1 feeding ratio in the synthesis. In contrast, A1‐PtPdFe‐31 demonstrated a different nucleation behavior. At 180 °C, only Pt nucleation was observed, forming ultrafine NPs (≈1.8 nm, Figure S3a,b, Supporting Information). Upon increasing the reaction temperature slightly to 190 °C, a complete reduction of all metal precursors was achieved after a holding time of 3 h, yielding a final PGM/Fe ratio of 76.4/23.6, with a slightly larger particle size of >5 nm (Figure S3c,d, Supporting Information). High‐resolution TEM analysis revealed that the (111) and (200) lattice spacings of A1‐PtPdFe‐11 were shorter than those of A1‐PtPdFe‐31 (Figure S4, Supporting Information). This observation, consistent with XRD data, suggests a more compressed lattice in A1‐PtPdFe‐11 due to its higher Fe content. The shift in diffraction peaks supports the modification of the crystal lattice, confirming composition‐dependent structural modulation in these disordered ternary alloys.

**Figure 1:**
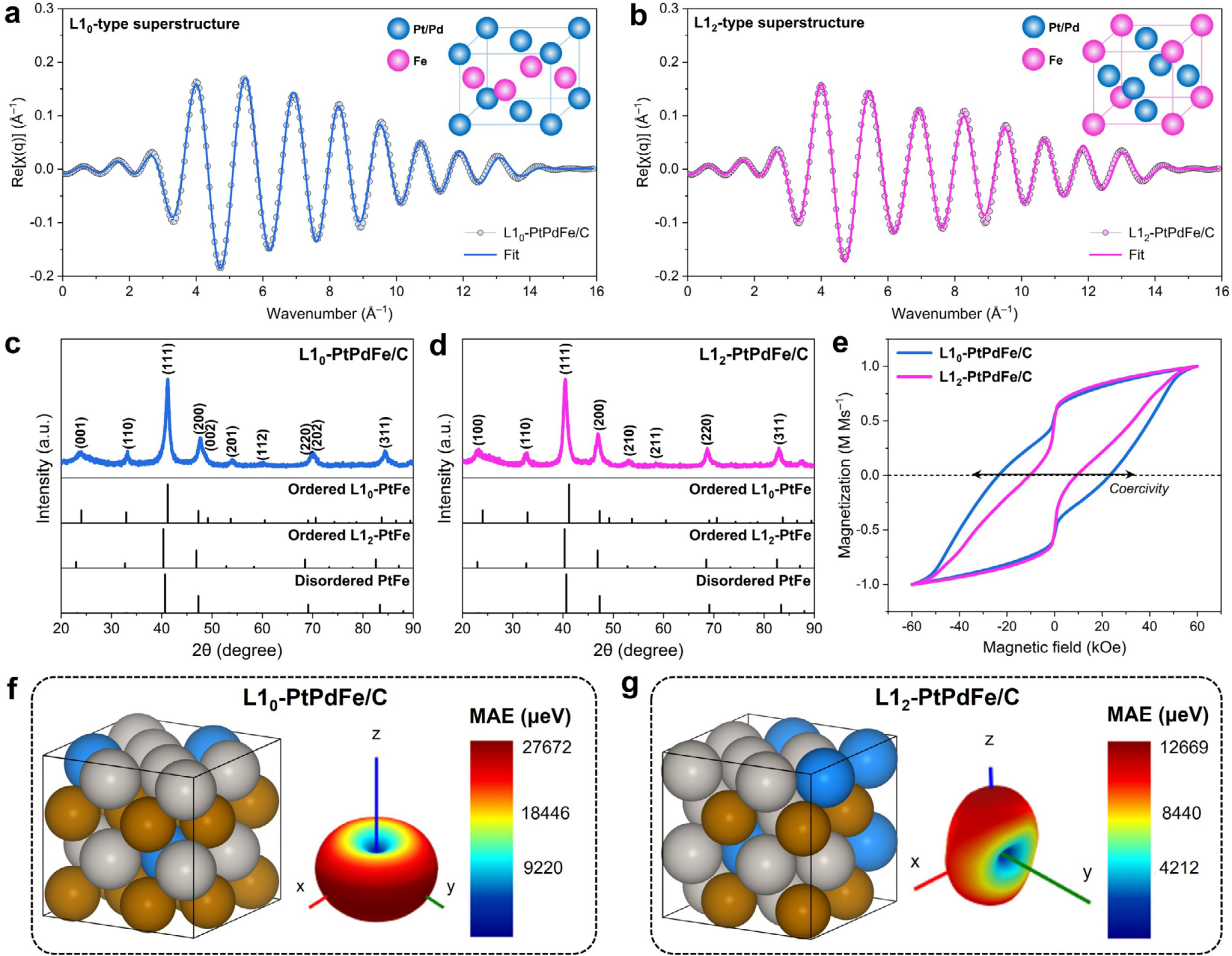
Structural characterizations of PtPdFe/C electrocatalysts. The *k*‐weighted EXAFS oscillations in *q*‐space of a) L1_0_‐PtPdFe/C and b) L1_2_‐PtPdFe/C for L1_0_‐ and L1_2_‐type superstructure fittings, respectively. XRD patterns of c) L1_0_‐PtPdFe/C and d) L1_2_‐PtPdFe MICs. The peaks are indexed by disordered cubic A1‐PtFe (PDF #03‐065‐9122), ordered tetragonal L1_0_‐PtFe (PDF #03‐065‐9121), and ordered cubic L1_2_‐PtFe (PDF #01‐089‐2050). e) Room‐temperature magnetic hysteresis (M–H) loops of L1_0_‐PtPdFe/C and L1_2_‐PtPdFe/C MICs. Image of the simulated bulk structure and the corresponding magnetic anisotropic energy plot for f) L1_0_‐PtPdFe/C and g) L1_2_‐PtPdFe/C MICs. Color codes: Pt (silver), Pd (blue), Fe (brown).

To induce ordering transformations, carbon‐supported A1‐PtPdFe/C NPs were subjected to thermal annealing, yielding L1_0_‐ and L1_2_‐type PtPdFe/C from A1‐PtPdFe‐11 and A1‐PtPdFe‐31, respectively. The extended X‐ray absorption fine structure (EXAFS) analysis, using scattering paths of standard tetragonal *P*4/*mmm* and cubic *Pm*‐3*m* PtFe structures, revealed well‐fitted k‐weighted oscillations for both L1_0_‐PtPdFe/C and L1_2_‐PtPdFe/C (Figure S5, Supporting Information), confirming the formation of ordered PtFe with distinct crystal structures (**Figure** **1**a,b). The EXAFS fitting parameters were summarized in Table S1 (Supporting Information). The results reveal that the Pt–Fe bond distance (d) in L1_0_‐PtPdFe/C is shorter than that in L1_2_‐PtPdFe/C, suggesting stronger 5d–3d orbital interactions in the L1_0_ phase. Additionally, the Pt–Fe coordination number (CN) is slightly higher in L1_0_‐PtPdFe, while the Pt–Pt(Pd) CN is higher in L1_2_‐PtPdFe. These results reflect a more anisotropic local bonding environment in L1_0_, consistent with its directional atomic ordering and higher degree of magnetocrystalline anisotropy, which will be discussed later. In contrast, the L1_2_‐PtPdFe structure features a more symmetric environment dominated by Pt–Pt(Pd) interactions. The XRD further verified these structural transformations (Figure 1c). The diffraction pattern of L1_0_‐PtPdFe/C exhibited characteristic superlattice peaks at 24.0°, 32.9°, 53.7°, and 60.5°, corresponding to the (001), (110), (201), and (112) ordering facets, respectively, of the fct L1_0_ intermetallic phase. The transformation of A1‐PtPdFe‐11/C to its L1_0_ can occur when the annealing temperature exceeds 600 °C, as indicated by the emergence of (001) and (110) superlattice reflections (Figure S6, Supporting Information). For further investigation, L1_0_‐PtPdFe/C annealed at 800 °C was selected due to its optimal ordering and crystallite size for ORR electrocatalysis (Table S2, Supporting Information). In comparison, the L1_2_‐PtPdFe/C exhibited superstructure reflections of (100), (110), (210), and (211) at 22.9°, 32.7°, 52.8°, and 58.3° (Figure 1d), respectively, which align with the standard card of fcc L1_2_‐type, differentiating the L1_2_‐PtPdFe/C from a former intermetallic phase. The disorder‐to‐order transition for L1_2_‐PtPdFe/C was visible at 700 °C (Figure S7, Supporting Information), as indicated by the appearance of superlattice peaks. Interestingly, during this transition stage, the major (111) peak gradually became sharper and shifted to lower angles with increasing temperature, indicating an expansion in lattice spacing and subsequent crystal size growth (Table S3, Supporting Information). Notably, the L1_2_‐PtPdFe/C annealed at 900 °C demonstrated the highest degree of atomic ordering and is therefore selected for further tests. To further confirm the critical role of Fe in driving the ordering transformation, we prepared PtPd alloys under the same conditions as those used for L1_0_‐PtPdFe/C and L1_2_‐PtPdFe/C but without Fe precursors. As shown in Figure S8 (Supporting Information), the XRD pattern of the resulting PtPd sample displays only peak characteristics of a disordered fcc PtPd structure, with no superlattice reflections observed. This contrasts sharply with the distinct superlattice peaks seen in the ternary PtPdFe systems (Figure 1c,d), which are indicative of long‐range atomic ordering. Atomic‐resolution high‐angle annular dark‐field scanning transmission electron microscopy (HAADF‐STEM) image further confirms that while Pt and Pd are indeed alloyed in the binary system, they do not form an ordered intermetallic phase (Figure S9, Supporting Information). This lack of ordering is likely due to the very small enthalpy of mixing between Pt and Pd, which provides little thermodynamic driving force for ordering.^[^ ^34^ ^]^ These results clearly demonstrate that ordering does not occur in the absence of Fe, highlighting its essential contribution to the formation of intermetallic phases in our PtPdFe ternary systems.

The formation of L1_0_ and L1_2_ intermetallic structures proceeds through a two‐stage process. Initially, the co‐reduction of Pt, Pd, and Fe precursors under solvothermal conditions results in kinetically driven nucleation and growth of disordered A1‐type alloy NPs. Upon thermal annealing at elevated temperature, atomic diffusion is facilitated, enabling the second stage of atomic rearrangement into thermodynamically stable ordered phases. The specific ordering pathway is strongly influenced by the atomic compositions.^[^ ^35^ ^]^ A near‐equiatomic PGM/Fe 1:1 ratio, as in A1‐PtPdFe‐11, provides the ideal site occupancy for tetragonal L1_0_ ordering, while a higher PGM‐to‐Fe ratio of 3:1, as in A1‐PtPdFe‐31, favors the stabilization of the cubic L1_2_‐type structure. After thermal annealing, both annealed L1_0_ and L1_2_ alloys were subjected to acid etching to remove unstable Fe and promote surface Pt enrichment. The Fe content of the annealed L1_0_‐PtPdFe/C and L1_2_‐PtPdFe/C remained unchanged prior to etching (Figure S10, Supporting Information), similar to their former A1 alloys, indicating no Fe loss during the thermal annealing step. Upon acid etching, however, the Fe leaching rate of 9–10% was observed for both samples. The Fe content in L1_0_‐PtPdFe/C rapidly stabilized at ≈40 at.% within 2 h, while L1_2_‐PtPdFe/C exhibited a more gradual decline, reaching ≈22 at.% after 6 h. These results suggest that the ordered alloys possess great chemical stability and resistance to Fe leaching, likely due to their structural robustness.

The magnetic properties of the MICs were investigated using a magnetic property measurement system (MPMS) at room temperature under an applied magnetic field of ±60 kOe. In the magnetic hysteresis (M–H) loops (Figure 1e), the L1_0_‐PtPdFe/C MIC exhibits a substantially higher coercivity of 23.8 kOe compared to 10.1 kOe for L1_2_‐PtPdFe/C. In contrast, the A1‐PtPdFe/C NPs displayed superparamagnetic behavior with weak yet nonzero magnetization (Figure S11, Supporting Information), confirming their lack of magnetic anisotropy in the disordered state. Such a pronounced magnetic difference can be further illustrated by the strong attraction of the MIC powders to an external magnet (Figure S12, Supporting Information). This significant difference in coercivity for the ordered L1_0_ phase indicates its strong uniaxial magnetic anisotropy. To further support the observed hard‐magnetic behavior, we calculated the effective magnetocrystalline anisotropy energy (MAE) using the coercivity‐based expression (Tables S4–S6, Supporting Information), which is accepted for nanoparticle system.^[^ ^36^, ^37^ ^]^ The experimental MAE density for L1_0_‐PtPdFe/C was 13.41 × 10^4^ J m^−3^ (Figure S13, Supporting Information), nearly twice that of L1_2_‐PtPdFe/C (7.58 × 10^4^ J m^−3^), confirming the superior magnetic anisotropy of the L1_0_ ternary alloy. Higher magnetic coercivity is widely recognized as a macroscopic indicator of large anisotropy energy, as it reflects the energy barrier for magnetization reversal, which directly arises from anisotropic spin‐orbit coupling in the crystal lattice.^[^ ^38^, ^39^, ^40^ ^]^

To complement our experimental findings in the magnetic properties of the ordered PtPdFe catalysts, we further calculated the magnetocrystalline anisotropy energy using first‐principles density functional theory (DFT) calculations (Figure 1f,g). The computed MAE for L1_0_‐PtPdFe/C was found to be approximately twice that of L1_2_‐PtPdFe/C, in excellent agreement with our experimentally calculated MAE. Calculated magnetic moments of the bulk crystals under magnetization directions are summarized in Table S7 (Supporting Information). The significantly higher MAE in L1_0_‐PtPdFe/C MICs can be attributed to the intrinsic symmetry differences, where L1_0_ intermetallics exhibit strong anisotropic spin‐orbit interactions due to their tetragonal structure, while L1_2_ cubic phases are magnetically more isotropic, which naturally leads to lower coercivity and thus reduced MAE. The alternating atomic layers within the unit cell (inset of Figure 1a) disrupt the cubic symmetry of the fcc structure, resulting in enhanced coercivity and magnetocrystalline anisotropy. To examine the origin of MAE enhancement, we performed comparative MAE calculations on Pt and PtPd alloys, which lack 5d–3d orbital hybridization. As shown in Figure S14 (Supporting Information), both systems exhibit negligible MAE values, in stark contrast to the significant MAEs obtained for the PtPdFe alloys. The low MAE in Pt and PtPd is likely due to the weaker orbital overlap and the absence of strong exchange interactions, as Pt and Pd contribute mainly paramagnetic character. This finding suggests that the incorporation of Fe 3d orbitals plays a critical role in inducing anisotropic magnetic behavior in these Pt‐based intermetallic catalysts.

**Figure 2:**
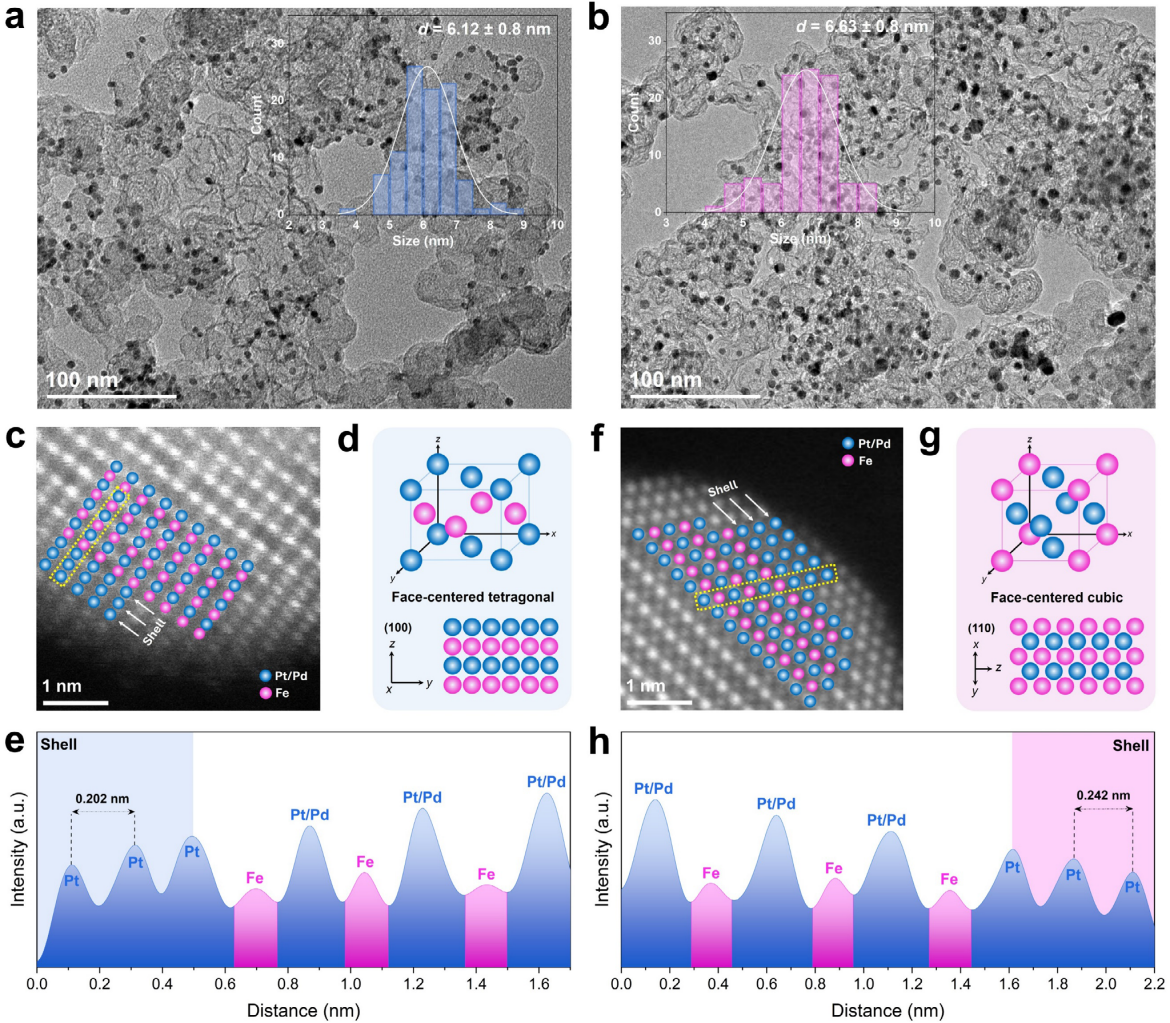
Morphological characterizations of PtPdFe/C electrocatalysts. TEM images of a) L1_0_‐PtPdFe/C and b) L1_2_‐PtPdFe/C and their corresponding particle size distributions. Atomic‐resolution HAADF‐STEM image, crystal structure, and corresponding line profile scanning analysis of c–e) L1_0_‐PtPdFe/C and f–h) L1_2_‐PtPdFe/C MICs.

The morphological characterizations of the MICs were conducted using TEM imaging. **Figure** **2**a,b illustrates a uniform distribution of well‐dispersed nanoparticles on the carbon support for both MICs, with no signs of agglomeration. The average particle size for L1_0_‐PtPdFe/C is 6.12 ± 0.8 nm, while that for L1_2_‐PtPdFe/C is slightly larger at 6.63 ± 0.8 nm, likely due to the higher concentration of PGM elements, which influence ordering kinetics and particle growth. In contrast, the PtFe samples, which were prepared under identical conditions such as those of L1_0_‐PtPdFe/C and L1_2_‐PtPdFe/C but without Pd precursors (Figure S15, Supporting Information), display agglomeration and broad size distribution (Figure S16, Supporting Information). The incorporation of Pd into the PtFe binary matrix was found to enhance thermal stability and prevent particle sintering by increasing configurational entropy and improving structural malleability, consistent with previous reports.^[^ ^41^, ^42^ ^]^ While compressive strain in ordered PtFe binary alloys may benefit ORR activity (Table S8, Supporting Information),^[^ ^43^, ^44^ ^]^ excessive lattice compression can destabilize surface atomic arrangements, promoting unfavorable Fe dissolution and local disordering during under acidic fuel cell conditions.^[^ ^45^, ^46^ ^]^ The ordered PtPdFe ternary alloys reveal the presence of well‐defined stacking sequences of ordered atomic columns, with Pt and Pd atoms appearing brighter than Fe due to their higher atomic number (*Z*) contrast (Figure 2c,f). This periodic arrangement provides strong evidence of intermetallic alloy formation in both MICs. Specifically, L1_0_‐PtPdFe/C exhibits a direct, periodic stacking of Pt (or Pd) and Fe columns along the [100] zone axis (Figure 2c,d), a characteristic feature of the L1_0_ ordered structure. Lattice spacing measurements of L1_0_‐PtPdFe/C particle indicate values of 0.378 and 0.291 nm, corresponding to the (001) and (110) superlattice planes, respectively (Figure S17a,b, Supporting Information). The interatomic distance between adjacent Pt/Pd and Fe atoms was determined to be ≈0.188 nm (Figure S17c, Supporting Information). On the other hand, L1_2_‐PtPdFe/C displays an alternating arrangement of atomic layers along the [110] facet, indicative of the L1_2_ ordered phase (Figure 2f,g). The measured lattice fringes were 0.392 and 0.288 nm, assigned to the (100) and (110) planes, respectively (Figure S18a,b, Supporting Information), with a neighboring atomic distance of ≈0.247 nm (Figure S18c, Supporting Information). Additionally, line intensity profiles obtained from Figure 2c,f illustrate an ordered core and 3 layers of shell region within the L1_0_‐PtPdFe/C and L1_2_‐PtPdFe/C MICs (Figure 2e,h). We also observed that the outermost Pt‐rich skin has a lattice spacing of 0.202 nm for L1_0_‐PtPdFe/C and 0.242 nm for L1_2_‐PtPdFe/C.

### 2.2 Fuel Cell Performance

The electrochemical evaluations were carried out for both MICs to explore how structural differences influence ORR activity. For comparison, the catalytic performance of a commercial Pt/C catalyst was also assessed under the same conditions. The metal loadings of the as‐prepared electrocatalysts on carbon support were analyzed by thermogravimetric analysis (TGA) and ICP‐OES. As shown in Figure S19 (Supporting Information), the total metal contents (Pt, Pd, and Fe) for L1_0_‐PtPdFe/C and L1_2_‐PtPdFe/C were determined to be 22.8 and 25.1 wt.%, respectively, which are comparable to that of commercial Pt/C (19.6 wt.%). Furthermore, ICP‐OES analysis revealed that the specific PGM (Pt + Pd) loadings were 19.0 wt.% for L1_0_‐PtPdFe/C and 22.8 wt.% for L1_2_‐PtPdFe/C, as summarized in Table S9 (Supporting Information). The ORR performance was first evaluated in a half‐cell setup using a rotating disk electrode (RDE) in 0.1 m HClO_4_ acidic electrolyte to obtain the polarization curves (Figure S20a, Supporting Information). The mass activity (MA) at 0.9 V was calculated to be 0.27, 0.89, and 0.56 A mg_PGM_^−1^ for commercial Pt/C, L1_0_‐PtPdFe/C, and L1_2_‐PtPdFe/C, respectively (Figure S20b, Supporting Information). These results indicate that alloying and ordering of Pt can significantly improve ORR activity. The superior ORR performance of L1_0_‐PtPdFe/C, as reflected in its more positive half‐wave potential and higher MA, suggests its advantages over L1_2_‐PtPdFe/C. To assess the ORR pathway, rotating ring‐disk electrode (RRDE) experiments were conducted in O_2_‐saturated 0.1 m HClO_4_ at room temperature (Figure S21, Supporting Information). The L1_0_‐PtPdFe/C and L1_2_‐PtPdFe/C MICs exhibited average electron transfer number (*n*) values of 3.99 and 3.99, respectively, indicating an efficient 4‐electron reduction pathway, similar to that of commercial Pt/C (3.97). Notably, L1_0_‐PtPdFe/C showed the lowest H_2_O_2_ yield (0.15%), followed by L1_2_‐PtPdFe/C (0.25%), both outperforming commercial Pt/C (0.52%). These results confirm that the PtPdFe MICs suppress undesired H_2_O_2_ formation, consistent with previous findings that Pt‐based catalysts can serve as radical scavengers and mitigate peroxide accumulation.^[^ ^47^, ^48^ ^]^

**Figure 3:**
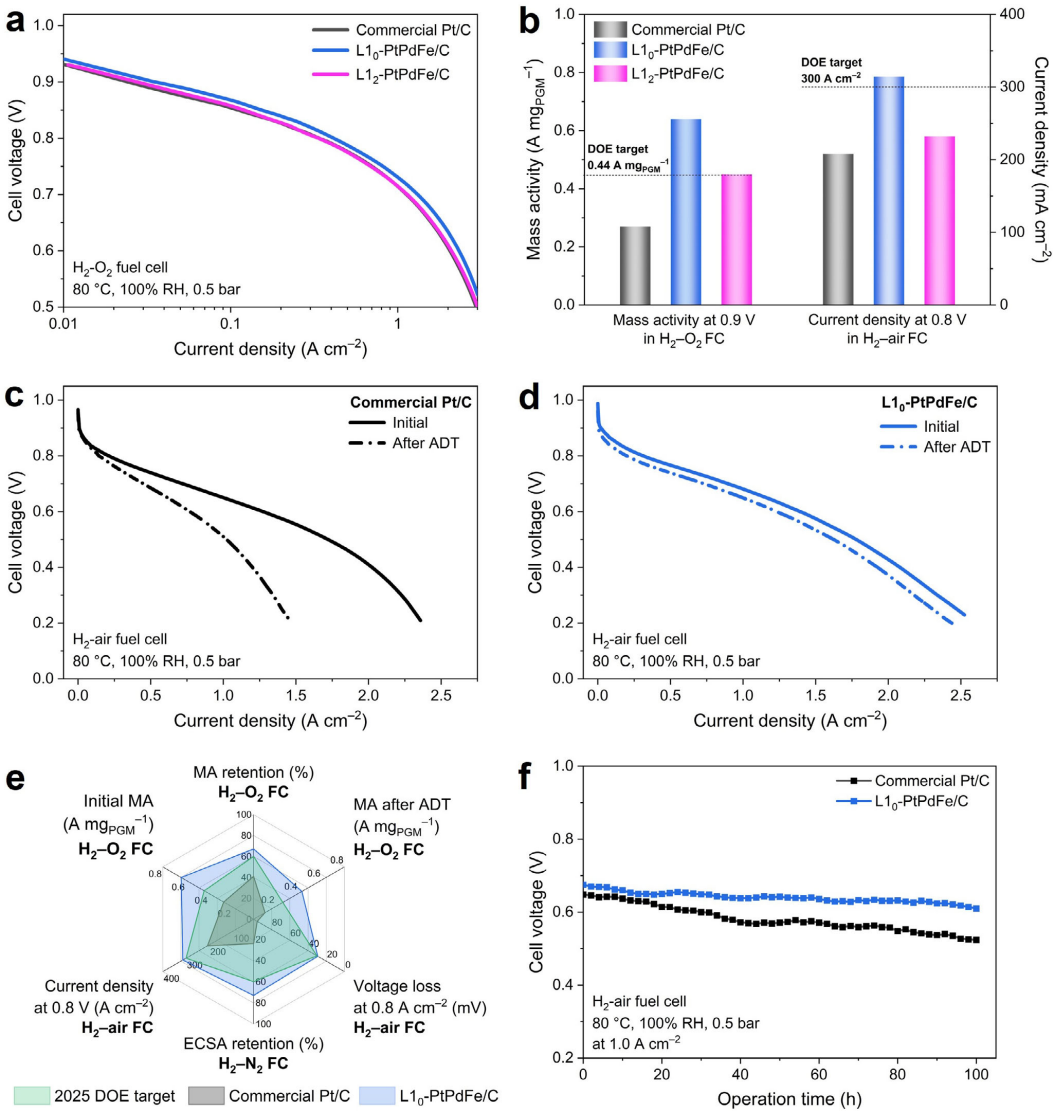
Electrocatalytic fuel cell performance. a) Fuel cell polarization curves recorded in H_2_–O_2_ condition assembled with cathode catalysts of commercial Pt/C, L1_0_‐PtPdFe/C, and L1_2_‐PtPdFe/C. b) Mass activity at 0.9 V and current density at 0.8 V of commercial Pt/C, L1_0_‐PtPdFe/C, and L1_2_‐PtPdFe/C under H_2_–O_2_ and H_2_–air conditions, respectively. H_2_–air polarization curves of c) commercial Pt/C and d) L1_0_‐PtPdFe/C MICs at initial and after 30 000 ADT cycles. e) Graphical achievement of fuel cell performance in H_2_–O_2_, H_2_–N_2_, and H_2_–air conditions for commercial Pt/C and L1_0_‐PtPdFe/C MICs compared to the 2025 DOE target. f) Steady‐state test at a constant current density of 1.0 A cm^−2^ for commercial Pt/C and L1_0_‐PtPdFe/C in H_2_–air. Test conditions: 80 °C, 100% relative humidity, 150 kPa_abs_ back pressure for H_2_/O_2_ and H_2_/air fuel cells. Gas flow rates: H_2_/O_2_ = 300/1000 cm^3^ min^−1^, H_2_/air = 300/1000 cm^3^ min^−1^, H_2_/N_2_ = 100/50 cm^3^ min^−1^ (without back pressure). Anode loading: 0.05 mg_Pt_ cm^−2^. Cathode loading: 0.10 mg cm^−2^. Membrane: Nafion N211.

To further examine the practical applicability of these materials in real fuel cells, single‐cell tests were performed by fabricating the as‐prepared MICs as cathode materials in the membrane electrode assembly (MEA) (Figure S22, Supporting Information), which is the core component of PEMFC.^[^ ^49^, ^50^ ^]^ The fuel cell polarization curves at low current densities, as depicted in **Figure** **3**a, compare the performance of commercial Pt/C and MICs. Under H_2_–O_2_ conditions, the L1_0_‐ and L1_2_‐PtPdFe cathode catalysts achieved MAs of 0.64 and 0.45 A mg_PGM_^−1^ at 0.9 V, respectively, surpassing both the commercial Pt/C (0.27 A mg_PGM_^−1^) and the DOE target (0.44 A mg_PGM_^−1^). In realistic H_2_–air operation, only L1_0_‐PtPdFe/C exceeded the DOE benchmark of 300 A cm^−2^ at 0.8 V (Figure 3b). The enhanced fuel cell performances of L1_0_‐PtPdFe/C under both H_2_–O_2_ and H_2_–air conditions again highlight the advantages of its L1_0_‐type fct structure over the L1_2_ fcc configuration. To assess catalyst durability, we conducted an accelerated durability test (ADT) for up to 30 000 potential cycles between 0.60 and 0.95 V, following DOE‐recommended electrocatalyst stability protocols (Figure S23, Supporting Information). Compared to commercial Pt/C, the L1_0_‐PtPdFe/C MICs demonstrated remarkable stability in H_2_–air after ADT (Figure 3c,d). Specifically, the voltage loss of L1_0_‐PtPdFe/C at a current density of 0.8 A cm^−2^ was only 29 mV, significantly lower than the degradation observed in commercial Pt/C (Figure S24, Supporting Information). In the H_2_–O_2_ environment, the MA at 0.9 V decreases to 0.43 A mg_PGM_^−1^ after 30 000 cycles, which corresponds to a 32.8% decrease (Figure S25, Supporting Information). The cyclic voltammetry (CV) results further confirmed that L1_0_‐PtPdFe/C maintained its electrochemical characteristics in H_2_–N_2_ (Figure S26, Supporting Information). The calculated electrochemically active surface area (ECSA) declines by 26.5% from 56.4 to 41.4 m^2^ g_Pt_^−1^, whereas commercial Pt/C exhibits a much more severe ECSA loss of 75.6%. Following these stability assessments in various conditions, the MEA fabricated by L1_0_‐PtPdFe/C cathode catalyst could retain its fuel cell performance beyond the 2025 DOE durability criteria (Figure 3e). We benchmarked our L1_0_‐PtPdFe/C catalyst in similar MEA test condition against representative Pt‐based catalysts reported in the literature, as summarized in Table S10 (Supporting Information). Our MIC demonstrates competitive fuel cell performance across various operational environments (H_2_–O_2_, H_2_–N_2_, and H_2_–air), combining high catalytic activity with excellent durability. To further validate its long‐term operational stability in real‐world fuel cells, an MEA using L1_0_‐PtPdFe/C MICs was continuously operated at a constant current density of 1.0 A cm^−2^ under H_2_–air for over 100 h, maintaining stable performance without severe degradation (Figure 3f). In contrast, the commercial Pt/C‐based MEA suffered an 18% decay in cell voltage over the same operating time.

Post‐stability TEM analysis revealed that the morphology of commercial Pt/C exhibited pronounced particle agglomeration following long‐term durability operation, with particle size increasing from 2.58 ± 0.7 nm in the initial test to 5.07 ± 1.4 nm after ADT (Figure S27, Supporting Information). This caused a significant reduction in ECSA during ADT, and thereby substantially decreasing its initial MA. Conversely, that of L1_0_‐PtPdFe/C MICs was well preserved, with no significant change in particle size distribution (Figure S28, Supporting Information). The average particle size of L1_0_‐PtPdFe/C was relatively stable, with a marginal increase from 6.73 ± 1.3 nm to 7.47 ± 1.3 nm, indicating its superior resistance to degradation. The STEM coupled with energy‐dispersive X‐ray spectroscopy (EDS) confirmed that the Pt‐rich shell in L1_0_‐PtPdFe/C remained intact (Figure S29, Supporting Information), as evidenced by only a minimal decrease in Fe content after ADT (Table S11, Supporting Information), suggesting less base metal leaching during electrochemical reactions. Furthermore, the HAADF‐STEM image verified the retention of the ordered structure in L1_0_‐PtPdFe/C post‐ADT (Figure S30, Supporting Information). Maintaining the structural integrity of electrocatalysts after prolonged operation is essential for sustaining high performance in ORR.^[^ ^51^ ^]^ High‐resolution XPS analysis further indicated that the Pt 4f, Pd 3d, and Fe 2p surfaces of L1_0_‐PtPdFe/C remained predominantly in the metallic state after ADT (Figure S31, Supporting Information). While a slight increase in oxidized species signals was observed for all three metals, this change is minimal, most likely due to mild surface oxidation after extended exposure to oxygen during ORR electrocatalysis. To assess durability at the magnetic level, M–H loops were measured using MPMS magnetometry along the out‐of‐plane direction on catalyst‐coated membranes (CCMs) extracted from MEA before and after ADT. As shown in Figure S32 (Supporting Information), both samples exhibited well‐defined ferromagnetic hysteresis. Notably, the coercivity of L1_0_‐PtPdFe/C decreased only from 9.1 to 8.4 kOe after ADT, indicating that the ferromagnetic ordering and magnetocrystalline anisotropy energy were largely preserved throughout the prolonged electrochemical test. This sustained coercivity reflects the robustness of directional 5d–3d orbital hybridization in the tetragonal L1_0_ structure, which supports spin‐polarized charge transport during ORR operation. These post‐ADT characterization results collectively confirm the exceptional structural stability of L1_0_‐PtPdFe/C, highlighting its potential as a durable ORR catalyst for PEMFC applications.

### 2.3 Theoretical and Spectroscopy Analysis

**Figure 4:**
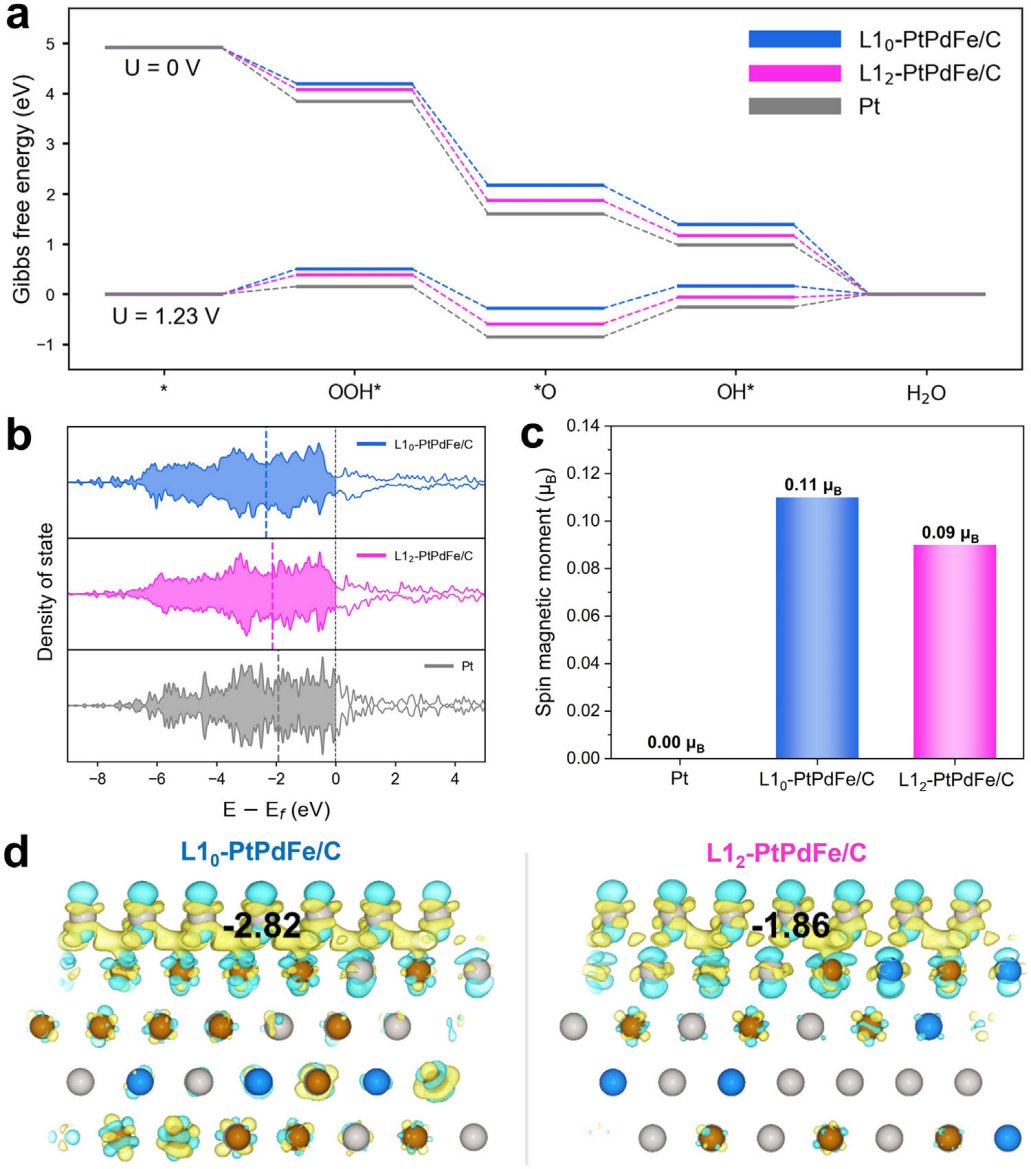
Theoretical analysis of the catalytic enhancement of the magnetic‐intermetallic catalyst. a) The Gibbs free energy diagram of L1_0_‐PtPdFe/C, L1_2_‐PtPdFe/C, and Pt at 0 and 1.23 V versus RHE. b) The projected density of states for the Pt layer in each model. The dotted line indicates the d‐band center. c) Spin magnetic moment calculated for Pt, L1_0_‐PtPdFe/C, and L1_2_‐PtPdFe/C. d) The charge density difference plots for L1_0_‐PtPdFe/C (left) and L1_2_‐PtPdFe/C (right). The isosurface level was set to 0.04 e/Bohr^3^, with the electron accumulation (depletion) represented by yellow (cyan) regions. The value represented the sum of the Bader charges of Pt skin.

To demonstrate that ORR is regulated by atomic ordering and magnetic moment, we performed density functional theory (DFT) calculations. The (111) surface structures with the first layer composed of Pt on top of L1_0_‐PtPdFe or L1_2_‐PtPdFe cores were modelled to simulate the effect of changing core substrates (Figures S33 and S34, Supporting Information). We then constructed the Gibbs free energy diagram of ORR, considering three reaction intermediates (OOH*, O*, and OH*) (**Figure** **4**a).^[^ ^52^ ^]^ We observed that the adsorption energies of reaction intermediates become stronger in the order of L1_0_‐PtPdFe/C, L1_2_‐PtPdFe/C, and Pt. According to the ORR volcano plot, a moderate weakening of adsorption strength relative to Pt (111) could lead to better catalytic activity.^[^ ^53^ ^]^ Indeed, the Gibbs free energy diagram revealed that the theoretical limiting potentials are 0.722 V for L1_0_‐PtPdFe/C, 0.695 V for L1_2_‐PtPdFe/C, and 0.630 V for Pt, demonstrating an improvement in ORR catalytic activity by up to 0.1 V when using L1_0_‐PtPdFe/C core. Density of states (DOS) calculations revealed a notable change in the magnetic properties of the Pt skin layer, transitioning from a paramagnetic to a ferromagnetic state, as indicated by the shift in DOS profile from symmetric to asymmetric (Figure 4b). This was further supported by the increase in magnetic moments from 0.00 µB per atom (Pt) to 0.09 µB per atom (L1_2_‐PtPdFe/C) and to 0.11 µB per atom (L1_0_‐PtPdFe/C) (Figure 4c). This change in magnetic behavior originates from the spin pinning effect, whereby the spins of Pt layer become aligned due to proximity to the underlying ferromagnetic core.^[^ ^54^ ^]^ The resulting spin alignment generates an internal magnetic field, which can act as a spin‐selective channel for the ORR, facilitating the spin transition for reducing triplet O_2_ to singlet H_2_O.^[^ ^55^ ^]^ Further, this modulation of the electronic structure caused a downshift in the d‐band center, from –1.93 eV (Pt) to –2.12 eV (L1_2_‐PtPdFe/C) to –2.34 eV (L1_0_‐PtPdFe/C), which leads to a moderate weakening of adsorption strength and thereby improves activity (Figure 4b). This trend implies that stronger magnetic properties correlate with enhanced catalytic performance. The charge density difference plots further revealed that the extent of charge accumulation at the interface between Pt skin and the core was more continuous and pronounced for L1_0_‐PtPdFe/C than L1_2_‐PtPdFe/C, which contributes to improved electronic conductivity. The Bader charge analysis confirmed a greater charge transfer effect, with the Pt skin of L1_0_‐PtPdFe/C exhibiting a Bader charge of –0.18 |e| per Pt atom, compared to –0.12 |e| per Pt atom for L1_2_‐PtPdFe/C (Figure 4d). Moreover, the calculated work function decreased from 5.64 eV (Pt) to 5.52 eV (L1_2_‐PtPdFe/C) and to 5.37 eV (L1_0_‐PtPdFe/C), indicating that electron transfer to intermediates becomes energetically more favorable when the ferromagnetic core exists (Figure S35, Supporting Information). These computational results highlight the critical role of ferromagnetic ordering in modulating the Pt surface layer that would be conducive to optimizing the ORR catalytic performance.

To gain further insight into the reaction pathway, we performed in situ attenuated total reflection‐Fourier transform infrared (ATR‐FTIR) spectroscopy in O_2_‐saturated 0.1 m HClO_4_ electrolyte. As shown in Figure S36a,b (Supporting Information), commercial Pt/C exhibits a vibrational band at ≈1264 cm^−1^, which corresponds to the adsorbed OOH* on the catalyst surface,^[^ ^56^, ^57^ ^]^ with increasing intensity by decreasing the applied potentials. This suggests an associative 4‐electron ORR mechanism, wherein O_2_ is first protonated to form OOH* before O–O bond cleavage (Figure S36c, Supporting Information). In contrast, L1_0_‐PtPdFe/C displays no detectable OOH* signal across the same potential range, pointing to a direct dissociative 4‐electron pathway. This dissociative route involves immediate cleavage of the O–O bond upon O_2_ adsorption, bypassing the formation of OOH*. Such a pathway is advantageous for minimizing side reactions and enhancing catalyst stability under acidic conditions.^[^ ^58^ ^]^ Both commercial Pt/C and L1_0_‐PtPdFe/C show strong signals at ∼1457 cm^−1^, confirming an O–O bond cleavage that ensures a complete 4e^–^ reduction to H_2_O. Notably, this feature appeared at higher potentials in L1_0_‐PtPdFe/C, suggesting earlier O–O bond cleavage and faster reaction kinetics.

**Figure 5:**
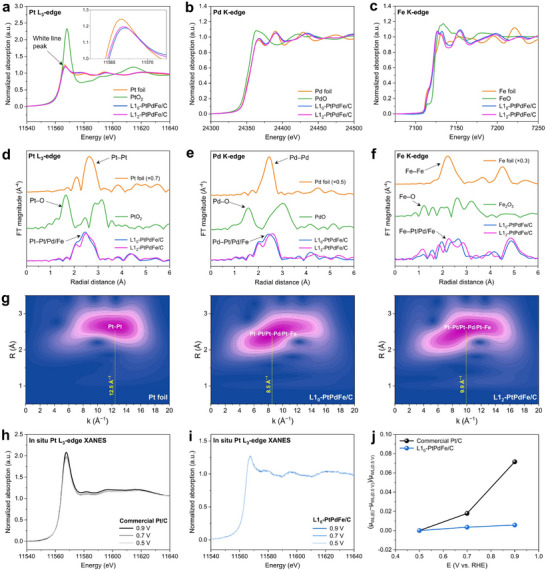
Spectroscopy analysis on the catalytic enhancement of the magnetic‐intermetallic catalyst. XANES and EXAFS spectra of L1_0_‐PtPdFe/C, L1_2_‐PtPdFe/C, foil, and oxide references at a,d) Pt L_3_‐edge, b,e) Pd K‐edge, and c,f) Fe K‐edge, respectively. g) Experimental Morlet wavelet transforms of k^3^‐weighted Pt L_3_‐edge for Pt foil, L1_0_‐PtPdFe/C, and L1_2_‐PtPdFe/C. In situ Pt L_3_‐edge XANES spectra of h) commercial Pt/C and i) L1_0_‐PtPdFe/C under applied potentials during ORR in O_2_‐saturated 0.1 m HClO_4_ electrolyte. j) Comparison of the change of normalized white line intensity (µ_WL_) of Pt L_3_‐edge XANES spectra relative to µ_WL_ at 0.5 V for commercial Pt/C and L1_0_‐PtPdFe/C as a function of applied potential.

To further investigate the surface electronic properties and coordination environment, we employed X‐ray absorption near‐edge structure (XANES) spectroscopy. The Pt L_3_‐edge XANES spectra of the MICs exhibit a lower normalized white line (WL) intensity compared to Pt foil and PtO_2_, with L1_0_‐PtPdFe/C displaying the lowest intensity (**Figure** **5**a). This suggests that Pt in the L1_0_ structure is stable in its metallic surface under open air. Similarly, the Pd K‐edge XANES spectra show a slightly increased WL intensity in MICs relative to Pd foil but significantly lower than PdO (Figure 5b), indicating that Pd in PtPdFe exists predominantly in a metallic state with minimal surface oxidation. In the normalized Fe K‐edge XANES spectra (Figure 5c), the absorption edges of the MICs lie between those of Fe foil and Fe_2_O_3_ but are closer to the metallic foil, suggesting that Fe remains mostly unoxidized. Notably, the Fe K‐edge XANES profile of the ternary MICs is obviously different from both Fe foil and Fe_2_O_3_, with a noticeable shift to lower energy. This shift likely arises from electronic modifications due to Fe insertion into the lattice of Pt and Pd, altering its local coordination environment. The extended X‐ray absorption fine structure (EXAFS) analysis provides further insights into the bonding characteristics. As illustrated in Figure 5d, the Pt L_3_‐edge EXAFS spectra of both MICs display a major peak between 2.0 and 3.0 Å, which represents the nearest coordination shells of the Pt atom assigned to Pt–Pt, Pt–Pd, and Pt–Fe interactions. This primary peak appears at a shorter radial distance than the Pt–Pt bond in Pt foil and PtO_2_, indicating bond length contraction due to alloying effects. Additionally, its lower intensity relative to Pt foil suggests a reduced first coordination number in PtPdFe MICs. A similar trend is also observed in the Pd K‐edge EXAFS spectra (Figure 5e), where the primary peak in L1_0_‐PtPdFe/C is positioned at a shorter distance than in L1_2_‐PtPdFe/C. This may indicate a stronger presence of ferromagnetic Fe in the L1_0_‐phase, as Pd–Fe bonds are shorter than Pd–Pd bonds. In good agreement with the Fe K‐edge XANES analysis, the main peak of MICs in the Fe K‐edge EXAFS spectra lies between the Fe–Fe bond in Fe foil and Fe_2_O_3_ (Figure 5f), suggesting a combination of various scattering paths, such as Fe–Fe path in Fe_2_O_3_ and Fe–Pt/Pd/Fe path in the PtPdFe ternary alloy. The experimental Morlet wavelet transform analysis at the k^3^‐weighted Pt L_3_‐edge verified that the Pt surface of the MICs was different from Pt foil (Figure 5g). Notably, their dominant signals appeared at shorter k and R values, indicative of scattering involving lighter elements such as Fe. The intensity maximum for L1_0_‐PtPdFe/C was centered at 8.5 Å^−1^, which is lower than that of L1_2_‐PtPdFe/C (9.9 Å^−1^) and Pt foil (12.5 Å^−1^). This suggests that strong ferromagnetic interactions in L1_0_‐PtPdFe/C, driven by Pt–Fe bonding, shorten the Pt–Pt distance of the surface. These spectral deviations from the corresponding metal foils indicate changes in the local coordination environments and electronic structure, suggesting strong orbital hybridization and charge redistribution between Pt and Fe atoms in the alloyed lattice. This 5d–3d orbital hybridization is known to enhance spin‐orbit coupling,^[^ ^59^ ^]^ which plays a critical role in determining the anisotropy energy of ferromagnetic intermetallic catalysts.

To understand the electrochemical stability of L1_0_‐PtPdFe/C MICs, we conducted in situ XANES at Pt L_3_‐edge during ORR in O_2_‐saturated 0.1 m HClO_4_ electrolyte. As shown in Figure 5h,i, the normalized white line intensity (µ_WL_) of Pt L_3_‐edge XANES spectra for L1_0_‐PtPdFe/C increases only slightly with increasing potential, while the commercial Pt/C displays a significant increase under the same working conditions. This difference becomes more apparent when comparing the relative change of µ_WL_ as a function of applied potential (Figure 5j). The minimal change in white line intensity with potential suggests a lower affinity for oxygen species chemisorption on Pt surface.^[^ ^60^, ^61^, ^62^ ^]^ This indicates that while O_2_ can adsorb effectively in L1_0_‐PtPdFe/C to initiate the ORR, the subsequent intermediates do not bind excessively, thereby facilitating rapid electrocatalysis. Furthermore, the increase in white line intensity with higher potential reflects the degree of Pt oxidation. While commercial Pt/C exhibits a pronounced increase, L1_0_‐PtPdFe/C shows only minor changes, suggesting enhanced surface electronic stability. This behavior underscores the superior electrochemical ORR robustness of the L1_0_‐PtPdFe/C MICs.

## 3 Conclusion

In summary, we report a ferromagnetic PtPdFe intermetallic alloy featuring a highly atomic ordering and magnetic characteristics. Comprehensive structural characterizations confirm the formation of different crystallographic phases of the alloy with L1_0_‐type tetragonal and L1_2_‐type cubic structures. The electrochemical evaluations reveal that L1_0_‐PtPdFe/C exhibits significantly superior ORR performance compared to its L1_2_ counterpart, which is attributed to its enhanced coercivity and greater magnetocrystalline anisotropy energy arising from ferromagnetic ordering of strong 5d–3d orbital interactions along the crystallographic c‐direction. The incorporation of Pd into PtFe alloys was found to suppress particle agglomeration, ensuring structural stability. Integrating Fe into PtPd facilitates the ordering transformation, investing the alloy with unique magnetic properties that play a crucial role in enhancing catalytic activity. Remarkably, after 30000 cycles of ADT, the L1_0_‐PtPdFe/C magnetic‐intermetallic catalyst retains its high performance with minimal degradation under required operating conditions under H_2_–O_2_, H_2_–air, and H_2_–N_2_. This work provides a profound insight into the intrinsic magnetic nature of ORR intermetallic catalysts and their potential for practical fuel cell applications.

## Conflict of Interest

The authors declare no conflict of interest.

## Author Contributions

M.I.M. performed conceptualization, methodology, investigation, validation, formal analysis, and wrote the original draft. J.K. performed methodology, investigation, validation, and formal analysis, and wrote the original draft. H.‐Y.L. and C.G.‐B. performed investigation, validation, and formal analysis. Y.W. and J.H.Y. performed investigation, validation, and formal analysis. J.H.S. and B.Y. performed validation, formal analysis. K.‐S.L. performed resources, validation; S.B. performed resources, funding acquisition, supervision, wrote, reviewed, and edited. J.‐S.Y. performed conceptualization, resources, funding acquisition, supervision, wrote, reviewed, and edited.

## Supporting information



Supporting Information

## Data Availability

The data supporting this article have been included as part of the Supporting Information.
