# Supplementary material for: Magnetocrystalline Anisotropic Platinum–Palladium–Iron Ternary Intermetallic Alloy for Enhanced Fuel Cell Electrocatalysis
Source: Adv Mater. 2025 Jul 28;37(41):e10314. doi: 10.1002/adma.202510314 (PMC12531720; doi:10.1002/adma.202510314)
Supplement: Supplementary file 1 — Supporting Information [file ADMA-37-e10314-s001.pdf]

ADVANCED MATERIALS

Supporting Information

for *Adv. Mater.*, DOI 10.1002/adma.202510314

Magnetocrystalline Anisotropic Platinum–Palladium–Iron Ternary Intermetallic Alloy for Enhanced Fuel Cell Electrocatalysis

Muhammad Irfansyah Maulana, Jungho Kim, Ha-Young Lee, Caleb Gyan-Barimah, Yi Wei, Jeong-Hoon Yu, Jong Hun Sung, Bo Yu, Kug-Seung Lee, Seoin Back and Jong-Sung Yu**

Supporting Information

Magnetocrystalline Anisotropic Platinum–Palladium–Iron Ternary Intermetallic Alloy for Enhanced Fuel Cell Electrocatalysis

Muhammad Irfansyah Maulana[1], Jungho Kim[2], Ha-Young Lee[3], Caleb Gyan-Barimah[1], Yi Wei[1], Jeong-Hoon Yu[1], Jong Hun Sung[1], Bo Yu[1], Kug-Seung Lee[4], Seoin Back[5,6,], and Jong-Sung Yu[1,3,*]*

1. Department of Energy Science and Engineering, Daegu Gyeongbuk Institute of Science and Technology (DGIST), Daegu, 42988, Republic of Korea
2. Department of Chemical and Biomolecular Engineering, Sogang University, Seoul, 04107, Republic of Korea
3. UE Science Inc., 66 Inan-gil, Gaejin-myeon, Goryeong-gun, Gyeongsangbuk-do, 40150, Republic of Korea
4. Pohang Accelerator Laboratory (PAL), Pohang University of Science and Technology (POSTECH), Pohang, 37673, Republic of Korea
5. KU-KIST Graduate School of Converging Science and Technology, Korea University, Seoul, 02841, Republic of Korea
6. Department of Integrative Energy Engineering, Korea University, Seoul, 02841, Republic of Korea

*Corresponding authors. E-mail: sback@korea.ac.kr (S. Back), jsyu@dgist.ac.kr (J.-S. Yu)

1. Experimental Section

1.1. Materials

Platinum(II) acetylacetonate (97%), palladium(II) acetylacetonate (99%), iron(III) acetylacetonate (97%), oleylamine (70%), oleic acid (99%), benzyl ether (99%), and perchloric acid (70%) were purchased from Sigma Aldrich. Ethanol (≥99.8%) and hexane (≥95.0%) were purchased from Samchun Chemical. Commercial carbon (Ketjen Black EC300JD) and Pt/C (TEC10E20A, 19.4 wt.% Pt loading) were purchased from Ketjen Black International and Tanaka Kikinzoku Kogyo, respectively. All chemicals were used as received.

1.2. Catalyst Preparation

The PtPdFe magnetic intermetallic catalysts (MICs) were first synthesized via a solvothermal reaction using platinum(II) acetylacetonate, palladium(II) acetylacetonate, and iron(III) acetylacetonate as metal precursors in a benzyl ether solution containing oleylamine and oleic acid. To achieve a metal loading of ~20%, a controlled amount of commercial carbon support was introduced into the solution. The precursor ratio of platinum group metal, PGM (Pt + Pd), to Fe was adjusted to 1:1 and 3:1 to form A1-PtPdFe-11/C and A1-PtPdFe-31/C nanoparticles (NPs), respectively, while maintaining a fixed Pt:Pd ratio of 3:1 in both NPs. The solution was extensively ultra-high sonicated for 2 h to ensure homogeneity, followed by solvothermal treatment at 200 °C for A1-PtPdFe-11/C and 190 °C for A1-PtPdFe-31/C for 5 h. After the reaction, the NPs were washed three times with a hexane/ethanol mixture and dried under an inert atmosphere. To induce the formation of ordered intermetallic structures, the A1-PtPdFe-11/C and A1-PtPdFe-31/C NP powders underwent thermal annealing under 5% H_2/Ar atmosphere at 800 °C for 6 h to form $L1_0$-PtPdFe/C and at 900 °C for 6 h to form $L1_2$-PtPdFe/C, respectively. The annealed powders were subsequently acid etched in 0.1 M $HClO_4$ at 60 °C for 12 h, followed by a final thermal treatment at 400 °C for 2 h to obtain the final catalysts.

1.3. Characterizations

The X-ray absorption spectroscopy (XAS) measurements were conducted at the Pohang Accelerator Laboratory (PAL) and analyzed using Demeter software package. The X-ray diffraction (XRD) analysis was performed on a Rigaku Smartlab diffractometer equipped with a Cu-Kα radiation source (λ = 1.5406 Å), operated at 40 kV and 30 mA. The X-ray photoelectron spectroscopy (XPS) spectra were acquired using an AXIS-NOVA instrument (Kratos) with a monochromated Al Kα X-ray source (hν = 1486.6 eV) operated at 150 W. The

magnetic property measurement system (MPMS) analysis was carried out using a Quantum Design MPMS3 system under an applied magnetic field of up to ±60 kOe. The transmission electron microscope (TEM) images were performed using an FEI Tecnai G2 F20, while the scanning transmission electron microscope (STEM) analysis, coupled with quantitative energy-dispersive X-ray spectroscopy (EDS), was conducted on an FEI Themis Z microscope. The thermogravimetric analysis (TGA) was carried out using a PerkinElmer Pyris instrument. The chemical composition of the catalysts was determined by inductively coupled plasma-optical emission spectrometry (ICP-OES) using a Thermo Scientific iCAP7400 system.

1.4. Electrochemical Evaluation

The half-cell electrochemical tests were performed using a rotating disk electrode (RDE) system connected to a Biologic VMP3 electrochemical analyzer. The working electrode was prepared by spray-coating a glassy carbon (GC) electrode with either commercial Pt/C or the as-prepared MICs. A calibrated Ag/AgCl electrode (saturated with KCl) and a Pt wire served as the reference and counter electrodes, respectively. To prepare the catalyst ink, 2.5 mg of the powder sample was dispersed in a 0.4 mL mixture of deionized water, isopropanol, and 0.5 wt.% Nafion solution. The dispersion was sonicated until a uniform suspension was obtained. A specific volume of the ink was then drop-cast onto the glassy carbon RDE (5 mm in diameter) to achieve a metal loading of 5 μg cm^{-2} for all catalysts. The oxygen reduction reaction (ORR) measurements were conducted in a 0.1 M $HClO_4$ solution saturated with either N_2 or O_2 gas. Prior to linear sweep voltammetry (LSV) measurements, the electrode was cycled 30 times in an N_2-saturated electrolyte at a scan rate of 50 mV s^{-1} to activate the catalysts. The LSV was subsequently recorded at a scan rate of 10 mV s^{-1} with an electrode rotation speed of 1600 rpm. All potentials were converted from Ag/AgCl to reversible hydrogen electrode (RHE) scale using the equation $E_{RHE} = E_{Ag/AgCl} + 0.256$. The peroxide yield ($H_2O_2$%) and electron transfer number (n) analyses were conducted using rotating ring-disk electrode (RRDE) Pt ring/GC disk electrode, and the values were calculated by the equation below:

$$\mathrm{H_2O_2}\ (\%) = 200 \times \frac{I_r/N}{I_d + I_r/N}$$

$$n = 4 \times \frac{I_d}{I_d + I_r/N}$$

where I_r, I_d, and N represent the ring current, disk current, and Pt ring current collection efficiency, respectively.

The membrane electrode assembly (MEA) for single-cell testing was fabricated using the catalyst-coated membrane (CCM) method with an active area of 5 cm^2. Commercial Pt/C and the as-prepared electrocatalysts were used as cathode catalysts, while commercial Pt/C was employed as the anode catalyst for all tests. The catalyst slurries were prepared by sonicating a mixture of catalyst powder, deionized water, 2-propanol, and a 5% Nafion ionomer solution (Sigma Aldrich) for 60 min. The water-to-2-propanol ratio was optimized at 3:7 (w/w), and the ionomer-to-carbon weight ratio at the cathode was maintained at 0.5. The metal loading was fixed at 0.10 mg cm^{-2} at the cathode and 0.05 mg cm^{-2} at the anode. The catalyst layer was directly sprayed onto a Nafion N211 membrane and dried at 60 °C for several hours before use. The MEA was assembled by sandwiching the CCM between two gas layers (GDLs, SGL 39BB) and two gaskets, which were pressed between graphite plates with a single-channel serpentine flow field (SCSFF) without hot-pressing.

The electrochemical single-cell tests were conducted using a polymer electrolyte membrane fuel cell (PEMFC) test station (Scitech Inc., Korea) and an electronic load (PLZ664WA, Kikusui). The polarization curve was recorded at 80 °C using fully humidified hydrogen (300 cm^3 min^{-1}) at the anode and oxygen (1000 cm^3 min^{-1}) or air (1000 cm^3 min^{-1}) at the cathode. The cyclic voltammetry (CV) measurements were performed at 80 °C without back pressure, where fully humidified hydrogen (100 cm^3 min^{-1}) and nitrogen (50 cm^3 min^{-1}) were supplied to the anode and cathode, respectively. The scan rate was set at 50 mV s^{-1} within a voltage window of 0.1–1.2 V. The electrochemically active surface area (ECSA) was determined by integrating the underpotential deposited hydrogen (H_{UPD}) peak, assuming a hydrogen adsorption charge of 210 μC cm_{Pt}^{-2}. During the test, the back pressure of the anode and cathode sections was kept at 150 kPa_{abs}. To evaluate the durability of the catalysts, accelerated durability test (ADT) was conducted based on US Department of Energy protocols. Electrocatalyst stability was assessed via square-wave voltage cycling between 0.6 and 0.95 V with a 3-sec hold at each potential, using fully humidified hydrogen (100 cm^3 min^{-1}) and nitrogen (50 cm^3 min^{-1}) at the anode and cathode, respectively, at 80 °C under ambient pressure. The MEA polarization curve and CV were recorded before and after ADT. Additionally, chronopotentiometry tests were performed at a constant current density of 1.0 A cm^{-2} for 100 h at 80 °C, with fully humidified hydrogen and air supplied to the anode and cathode at a flow rate of 300 and 1000 cm^3 min^{-1}, respectively, under a backpressure of 150 kPa_{abs}.

1.5. Theoretical Calculation

Density functional theory (DFT) calculations were performed using the Vienna Ab Initio Simulation Package (VASP) version 5.4.4.[1,2] The generalized gradient approximation (GGA) with the Perdew-Burke-Ernzerhof (PBE) exchange-correlational functional and projector augmented wave (PAW) pseudopotentials were used.[3–5] Noncollinear DFT calculations were employed to account for the spin-orbit coupling.[6] The DFT-D3 method by Grimme was included to consider van der Waals interactions in the system.[7,8] The cutoff energy for the plane-wave basis set was set to 400 eV. All calculations were considered converged when the energy and force became smaller than 10^{-4} eV and 0.05 eV/Å, respectively. Gamma-centered k-point grid ($4 \times 4 \times 4$) and ($3 \times 3 \times 1$) was sampled for bulk and surface models, respectively.[9] The bulk structures of $L1_0$ and $L1_2$-type ternary PtPdFe alloys were constructed using $2 \times 2 \times 2$ supercells, each consisting of 32 atoms. The lattice constants were set to their experimental values. To explore various atomic configurations, a total of 42 unique bulk configurations for $L1_0$ and 603 for $L1_2$ were identified using the bsym package.[10] The structure with the lowest DFT energy was selected through a screening process utilizing Crystal Graph Convolutional Neural Networks (CGCNN) subsequently validated by DFT calculations.[11] A 5-layered (111) surface structure was modeled based on the corresponding bulk structure (Figure S34, Supporting Information). The topmost layer was relaxed during optimization, while the other layers were fixed to their bulk positions. A vacuum layer of 20 Å was added in the z-direction. Following the $4e^-$ ORR pathway, in which four consecutive proton-electron transfers occur, the Gibbs free energies of each step were calculated by adding free energy corrections to DFT-calculated electronic energies. The correction term consisted of zero-point energies, enthalpic and entropic contributions. The computational hydrogen electrode (CHE) method was employed, which assumes that the chemical potential of proton-electron pair is equivalent to half that of hydrogen gas under standard conditions.[12] The magnetic anisotropy energy was calculated as the difference between the energies of the easiest and hardiest axis of magnetization, using the VASPKIT package.[13]

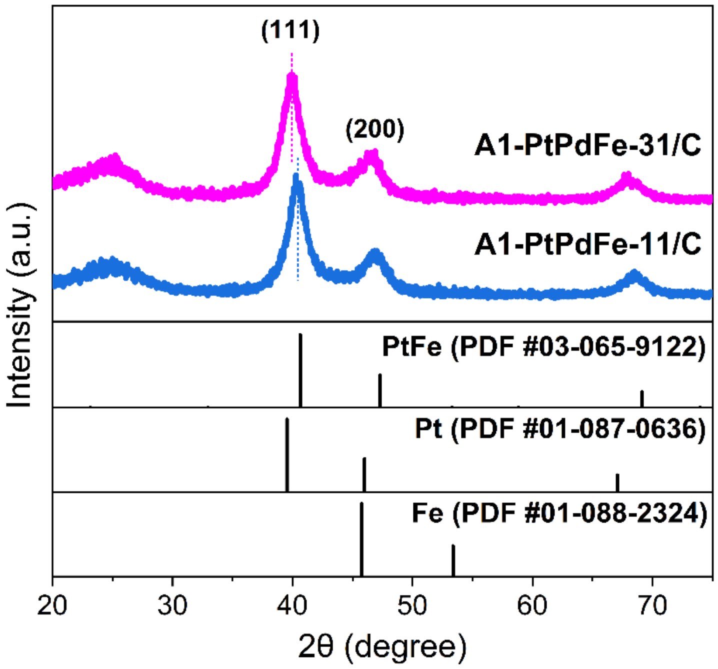

Figure S1. XRD patterns of A1-PtPdFe-11/C and A1-PtPdFe-31/C alloy NPs with different PGM/Fe mole ratios of 1:1 and 3:1, respectively. The XRD peaks are indexed by disordered PtFe (PDF #03–065–9122), Pt (PDF #01-087-0636), and Fe (PDF #01-088-2324).

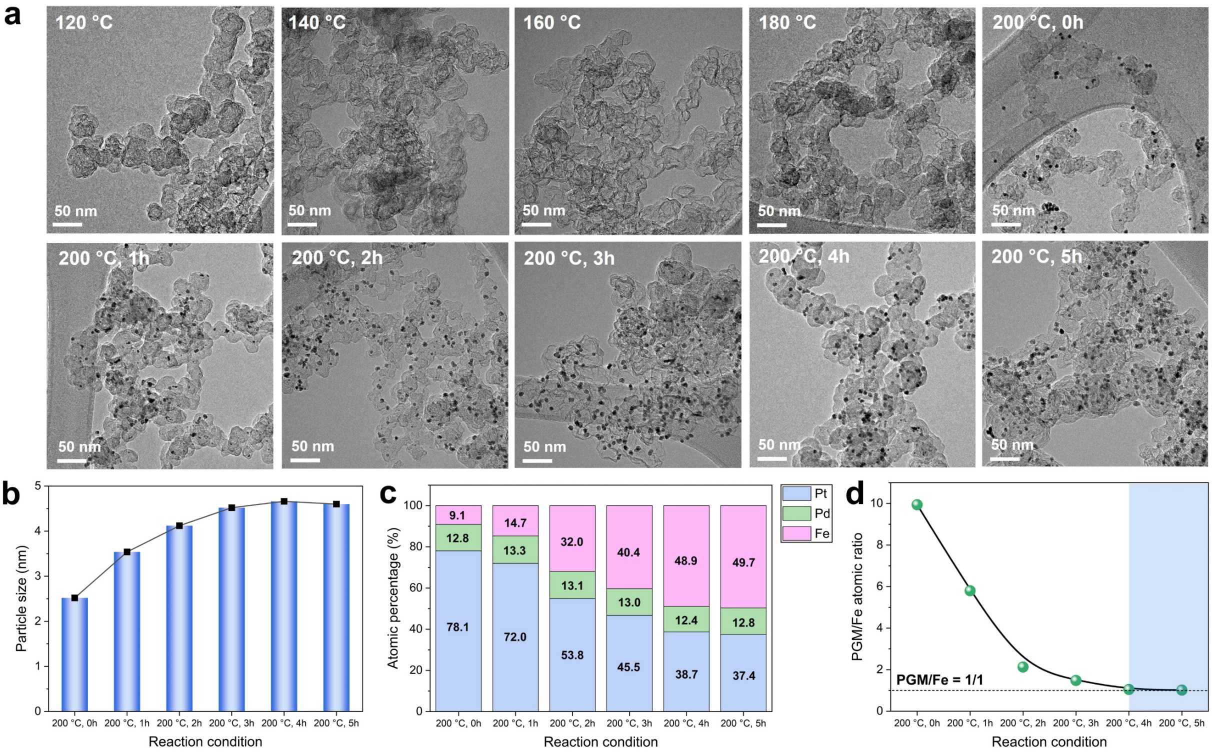

Figure S2. a) Ex situ TEM images during the alloying stage of A1-PtPdFe-11/C with the PGM/Fe mole ratio of 1:1 and b) corresponding average particle sizes at different reaction times. c) Atomic percentage of the A1-PtPdFe-11/C NPs and d) their experimental PGM/Fe ratio obtained by ICP-OES measurements.

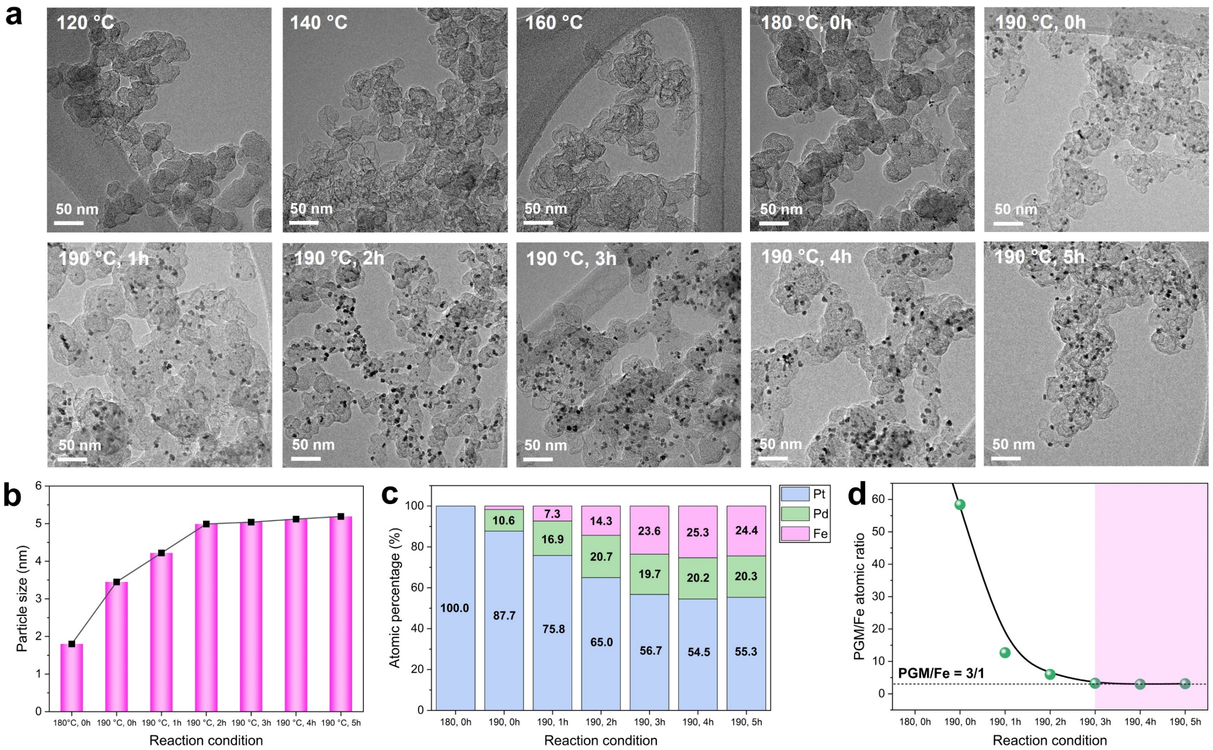

Figure S3. a) Ex situ TEM images during the alloying stage of A1-PtPdFe-31/C with the PGM/Fe mole ratio of 3:1 and b) corresponding average particle sizes at different reaction times. c) Atomic percentage of the A1-PtPdFe-31/C NPs and d) their experimental PGM/Fe ratio obtained by ICP-OES measurements.

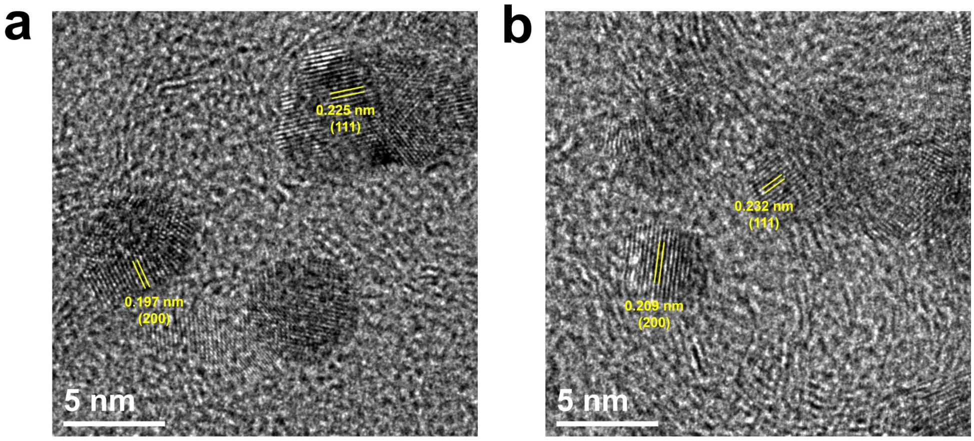

Figure S4. HR-TEM images of (a) A1-PtPdFe-11/C and (b) A1-PtPdFe-31/C NPs and their corresponding lattice fringe spacings.

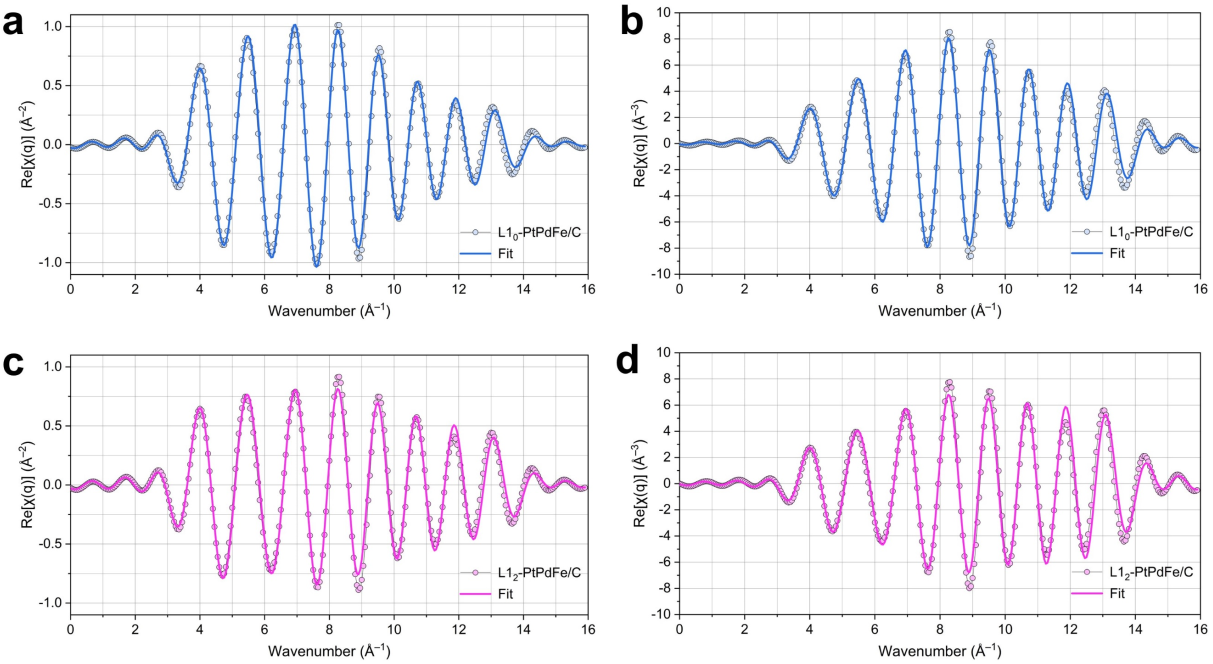

Figure S5. The EXAFS oscillations in *q*-space of a,b) $L1_0$-PtPdFe/C and c,d) $L1_2$-PtPdFe/C for $L1_0$- and $L1_2$-type superstructure fittings, respectively, with different k-weights.

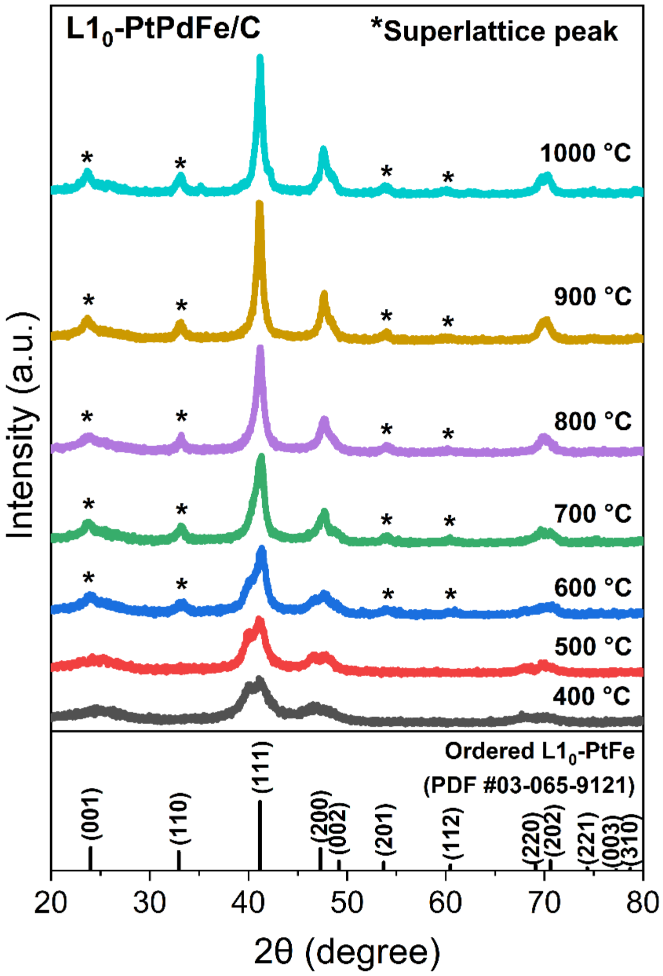

Figure S6. Ex situ XRD patterns of the obtained L1$_0$-PtPdFe/C transformed from A1-PtPdFe-11/C at different thermal annealing temperature from 400 to 1000 °C for a holding time of 6 h. The peaks are indexed by standard ordered L1$_0$-PtFe with fct structure (PDF #03-065-9121).

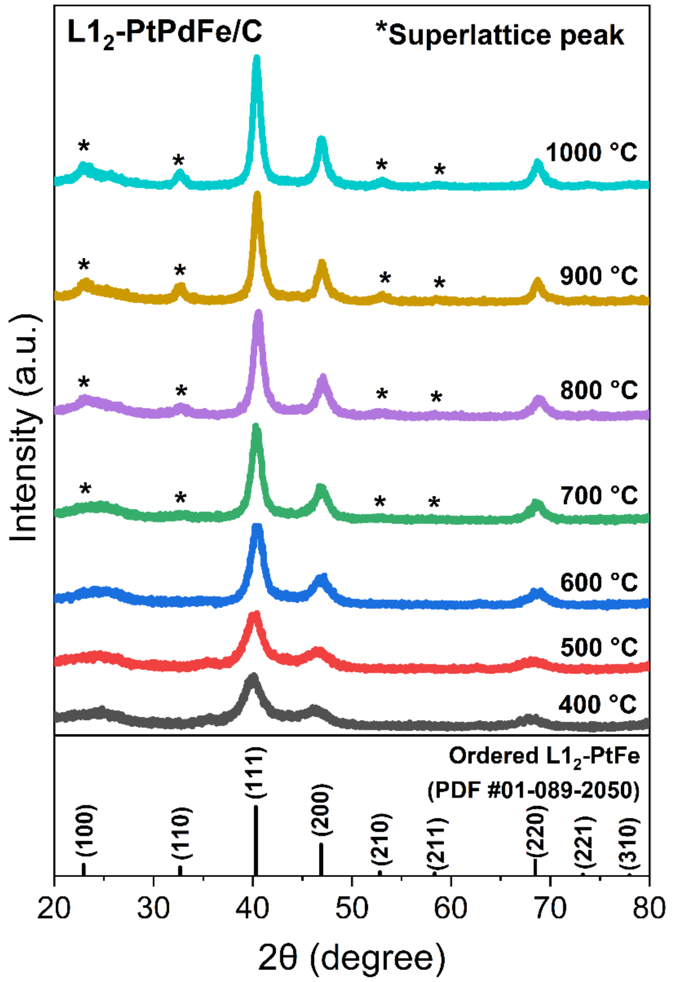

Figure S7. Ex situ XRD patterns of the obtained L1$_2$-PtPdFe/C transformed from A1-PtPdFe-31/C at different thermal annealing temperature from 400 to 1000 °C for a holding time of 6 h. The peaks are indexed by standard ordered L1$_2$-PtFe with fcc structure (PDF #01-089-2050).

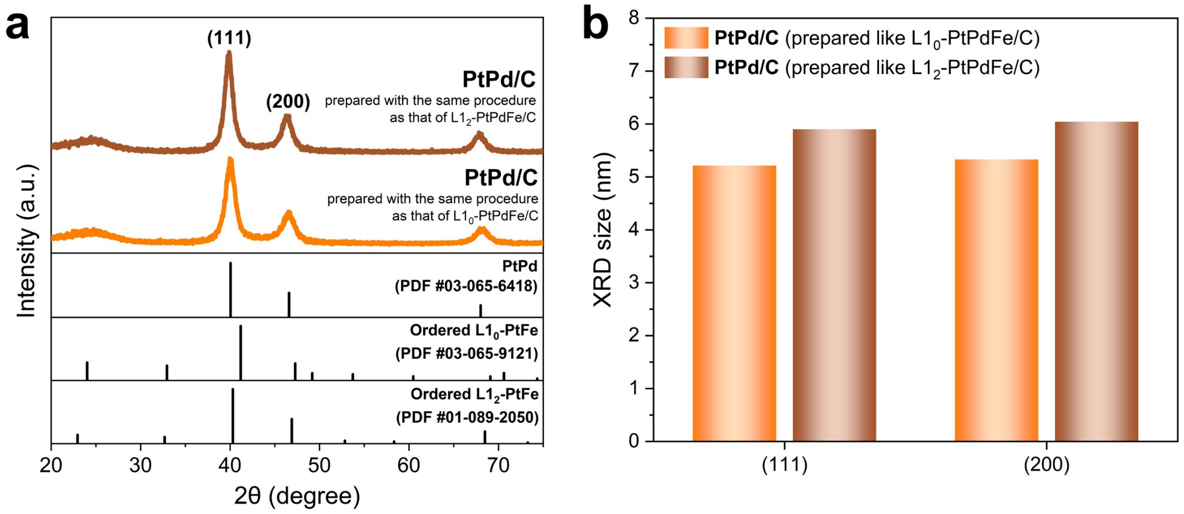

Figure S8. a) XRD patterns and b) corresponding crystallite sizes along the (111) and (200) facets of PtPd samples prepared with the same procedure as that of $L1_0$-PtPdFe/C and $L1_2$-PtPdFe/C without the addition of Fe precursors. The peaks are indexed by standard PtPd with fcc structure (PDF #03-065-6418), ordered $L1_0$-PtFe with fct structure (PDF #03-065-9121), and ordered $L1_2$-PtFe with fcc structure (PDF #01-089-2050). The XRD crystallite sizes were calculated using Debye–Scherrer equation.

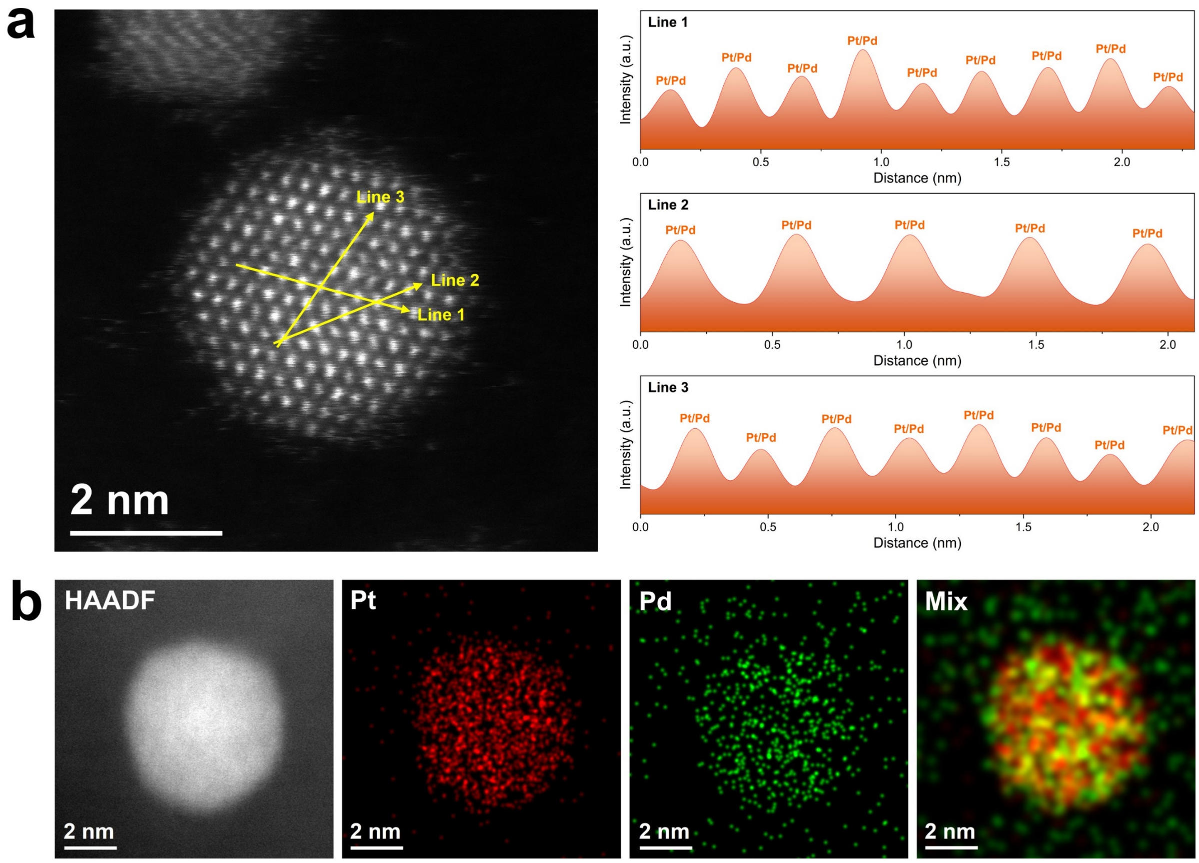

Figure S9. a) Atomic-resolution HAADF-STEM image and corresponding line profile analysis and b) STEM-EDS elemental mapping of a representative PtPd sample prepared with the same procedure as that of $L1_0$-PtPdFe/C without the addition of Fe precursors.

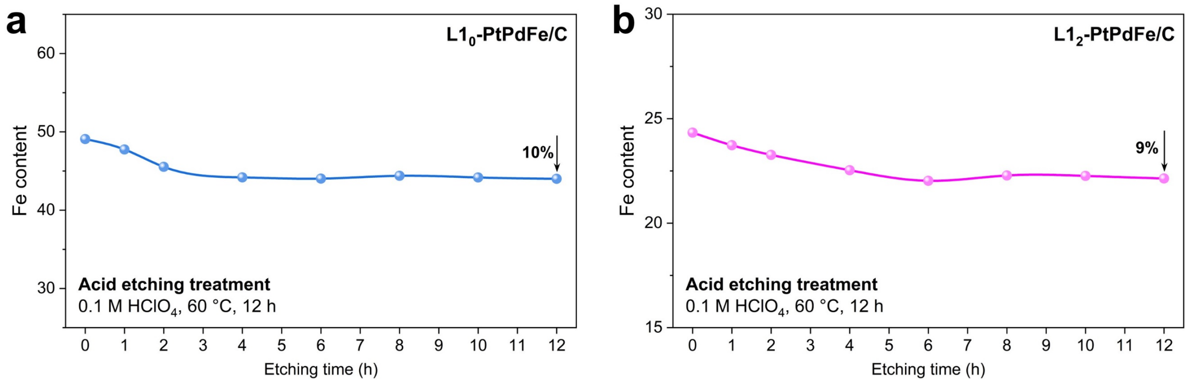

Figure S10. Time-resolved ICP-OES results of Fe content for a) $L1_0$-PtPdFe/C and b) $L1_2$-PtPdFe/C during 12 h of acid etching treatment in 0.1 M $HClO_4$ at 60 °C.

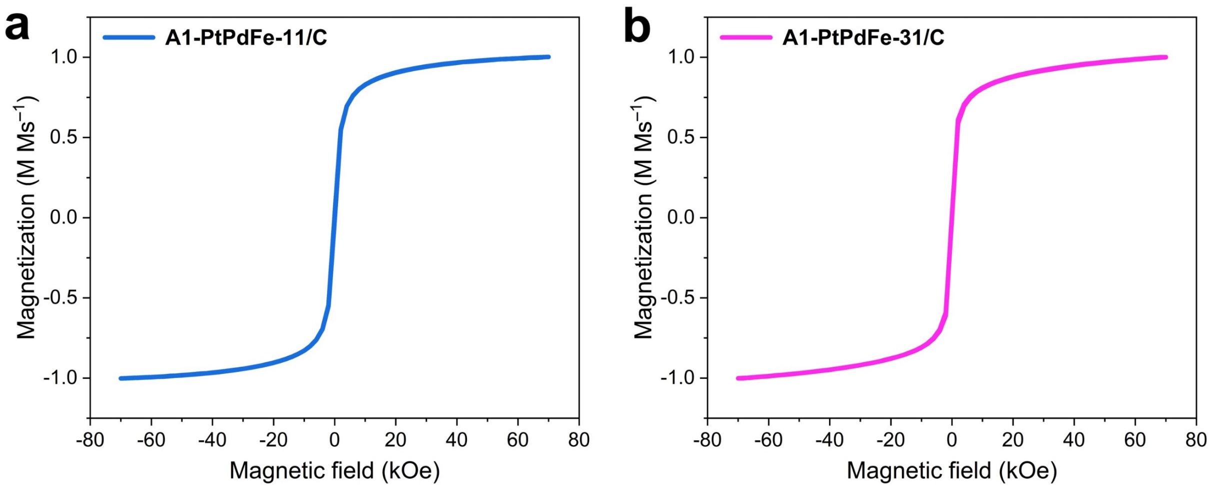

Figure S11. Magnetic hysteresis loops of a) A1-PtPdFe-11/C and b) A1-PtPdFe-31/C NPs.

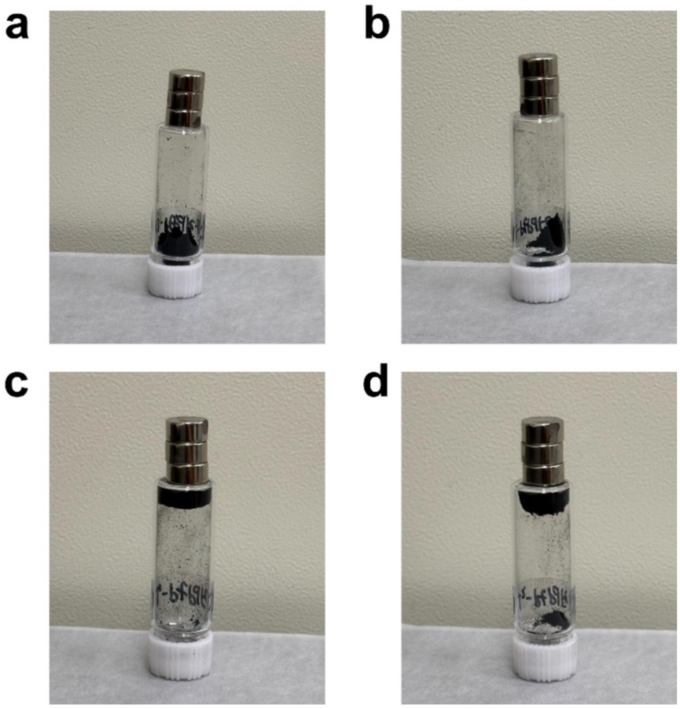

Figure S12. Photographs of the powder samples of a) A1-PtPdFe-11/C, b) A1-PtPdFe-31/C, c) $L1_0$-PtPdFe/C, and d) $L1_2$-PtPdFe/C, where an external magnet is placed outside the vial.

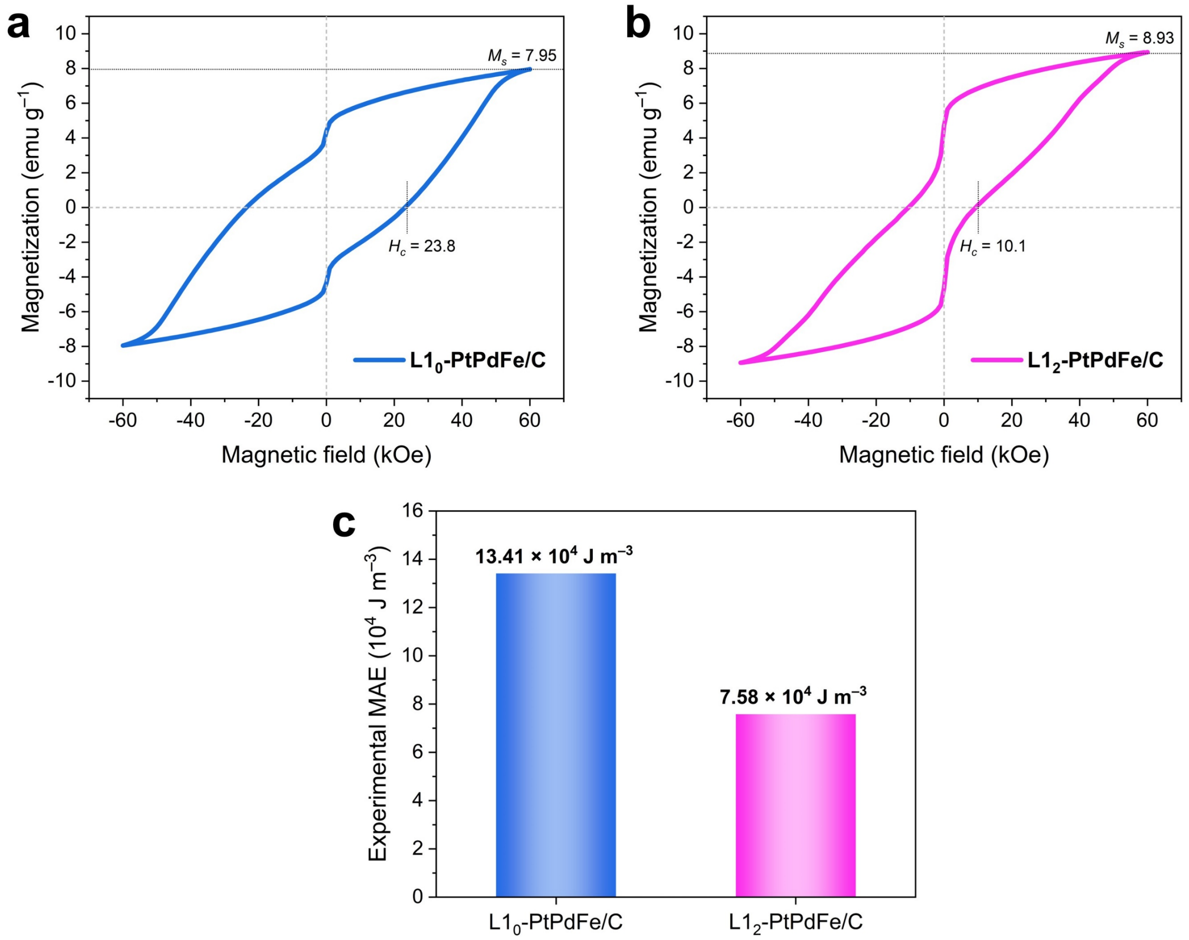

Figure S13. The coercivity (H_c) and saturation magnetization (M_s) values for a) L1$_0$-PtPdFe/C and b) L1$_2$-PtPdFe/C measured from magnetic hysteresis (M–H) loops. The H_c is expressed in kOe and M_s is in emu g^{-1}. c) Experimentally calculated magnetocrystalline anisotropy energy for L1$_0$-PtPdFe/C and L1$_2$-PtPdFe/C based on the extracted H_c and M_s values.

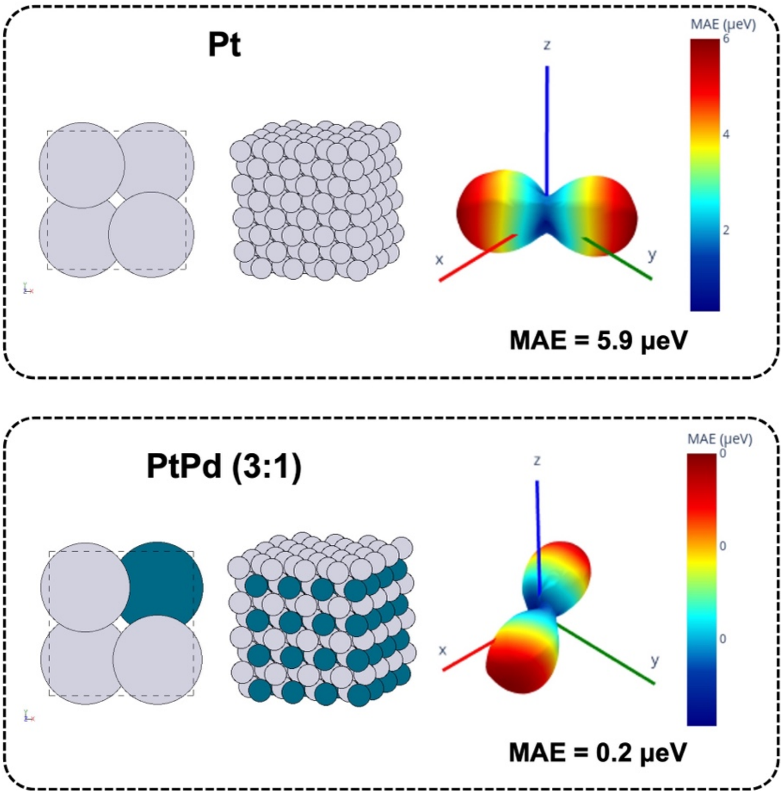

Figure S14. Image of the simulated bulk structure and the corresponding magnetic anisotropic energy plot for Pt and PtPd. Color codes: Pt (grey), Pd (blue green).

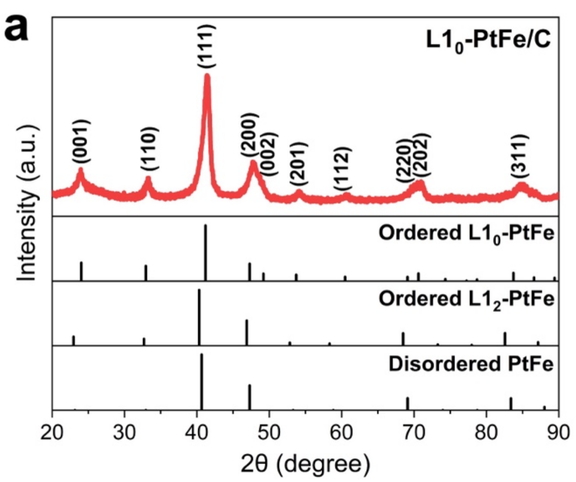

Figure S15. XRD patterns of PtFe samples prepared with the same procedure as that of a) L1$_0$-PtPdFe/C and b) L1$_2$-PtPdFe/C without the addition of Pd precursors. The peaks are indexed by disordered cubic A1-PtFe (PDF #03-065-9122), ordered tetragonal L1$_0$-PtFe (PDF #03-065-9121), and ordered cubic L1$_2$-PtFe (PDF #01-089-2050).

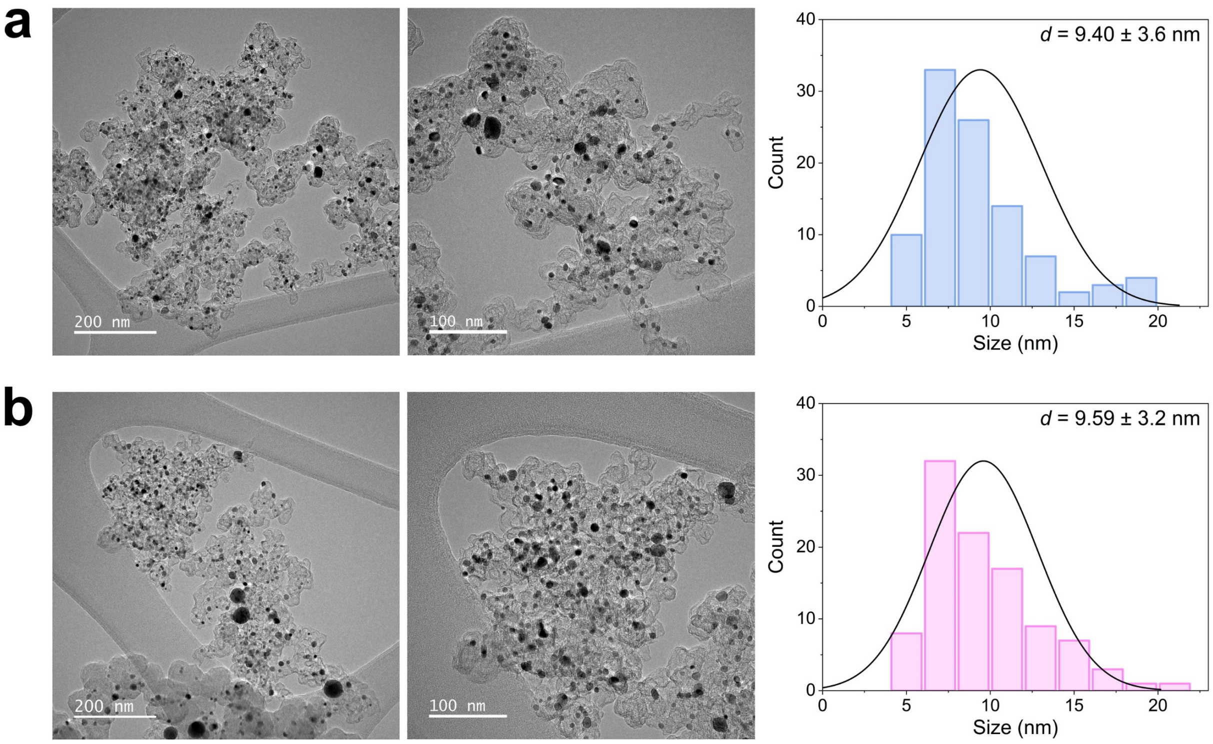

Figure S16. TEM images and particle distribution histogram of PtFe samples prepared with the same procedure as that of a) $L1_0$-PtPdFe/C and b) $L1_2$-PtPdFe/C without the addition of Pd precursors.

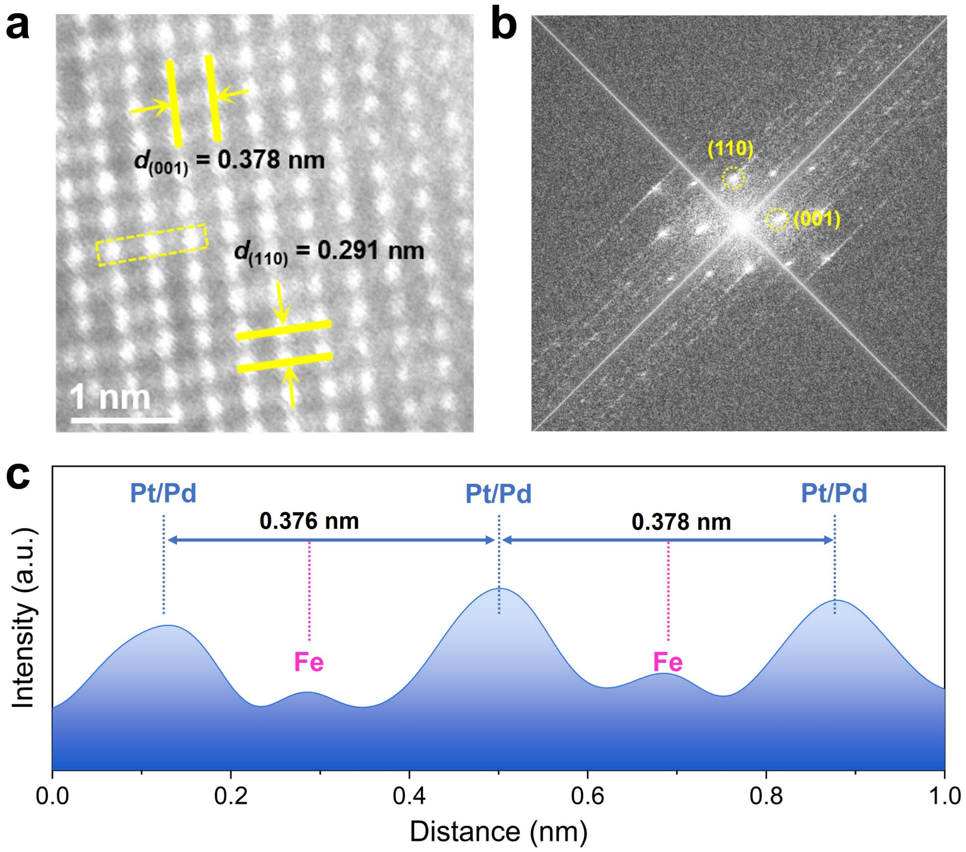

Figure S17. a) The atomic-resolution HAADF-STEM image of the ordered $L1_0$-PtPdFe/C particle and b) its corresponding FFT pattern. c) The line intensity profile of the $L1_0$-PtPdFe/C along the dashed yellow rectangle in (a).

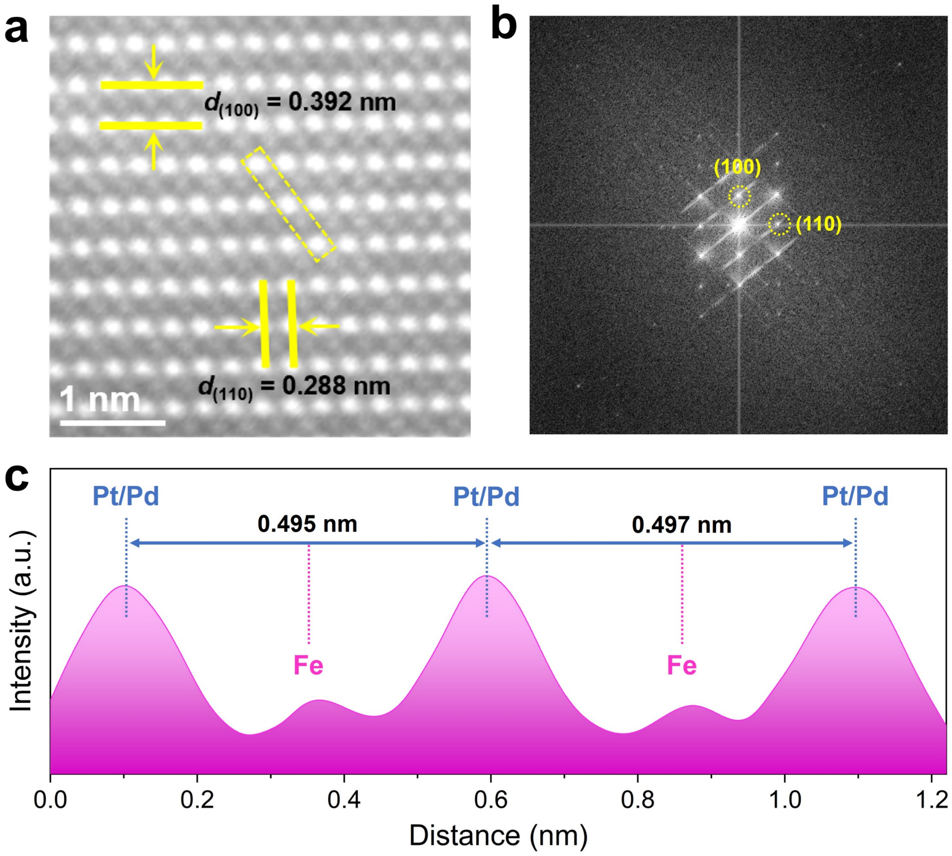

Figure S18. a) The atomic-resolution HAADF-STEM image of the ordered $L1_2$-PtPdFe/C particle and b) its corresponding FFT pattern. c) The line intensity profile of the $L1_2$-PtPdFe/C along the dashed yellow rectangle in (a).

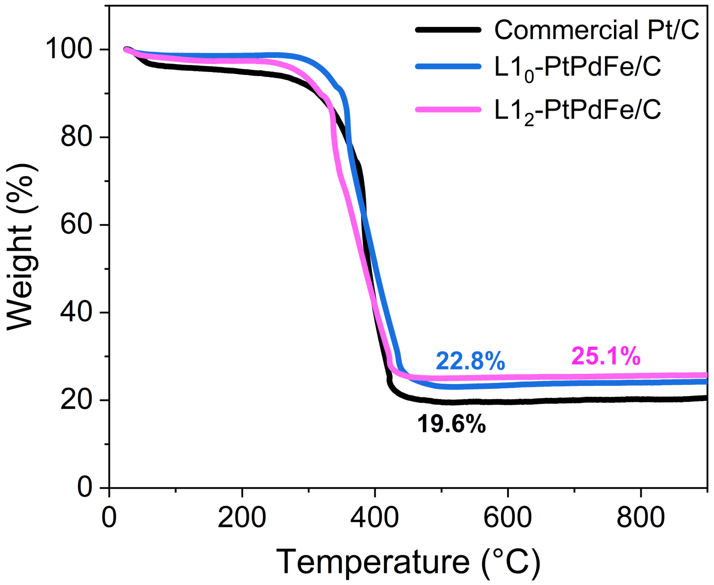

Figure S19. TGA curves of commercial Pt/C, L1$_0$-PtPdFe/C, and L1$_2$-PtPdFe/C in air condition with the indication of their metal weight loadings.

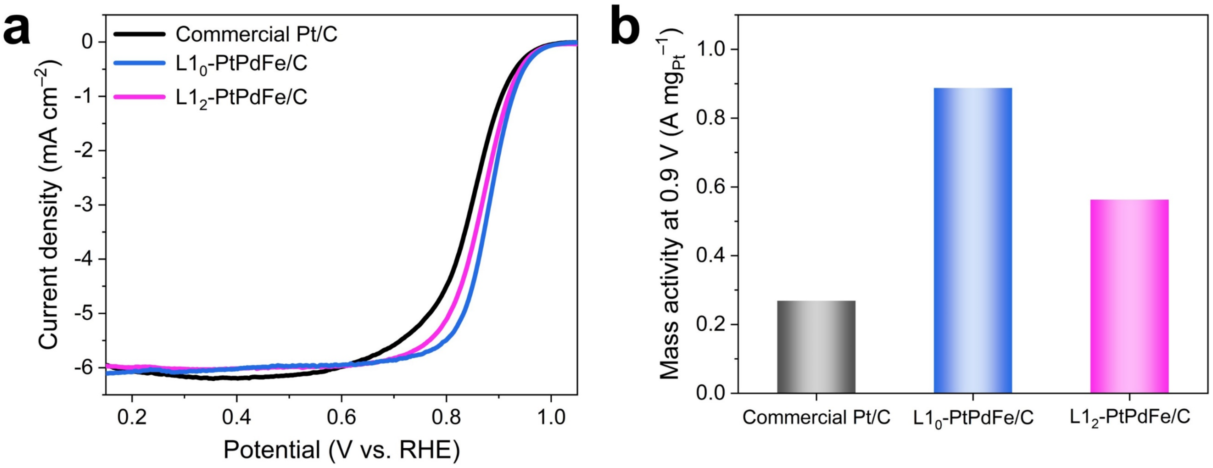

Figure S20. a) ORR polarization curves and b) corresponding mass activity of commercial Pt/C, $L1_0$-PtPdFe/C, and $L1_2$-PtPdFe/C in 0.1 M $HClO_4$.

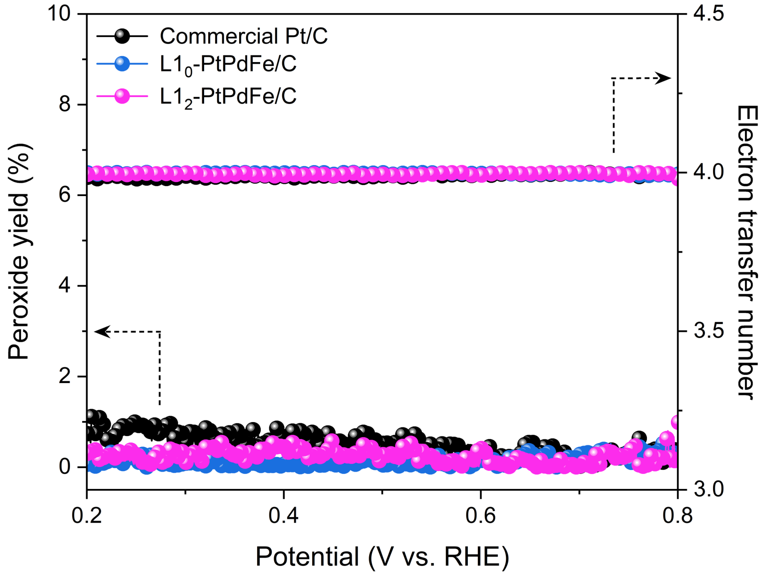

Figure S21. Peroxide yields and electron transfer numbers of commercial Pt/C, L1$_0$-PtPdFe/C, and L1$_2$-PtPdFe/C MICs in O_2-saturated 0.1 M $HClO_4$ electrolyte determined by RRDE test.

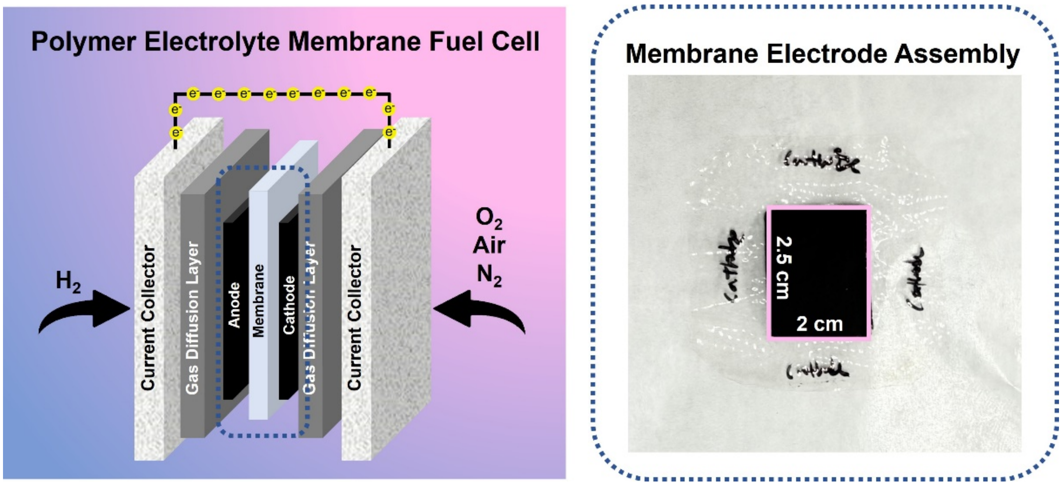

Figure S22. The schematic illustration of a single PEMFC, where the polymer electrolyte membrane (PEM) and catalyst layers (CLs) form a membrane electrode assembly (MEA).

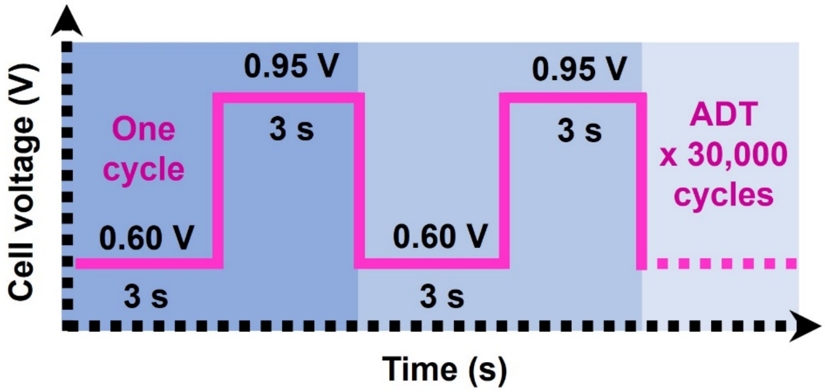

Figure S23. Accelerated durability test (ADT) protocols for ORR catalyst evaluation set by DOE.

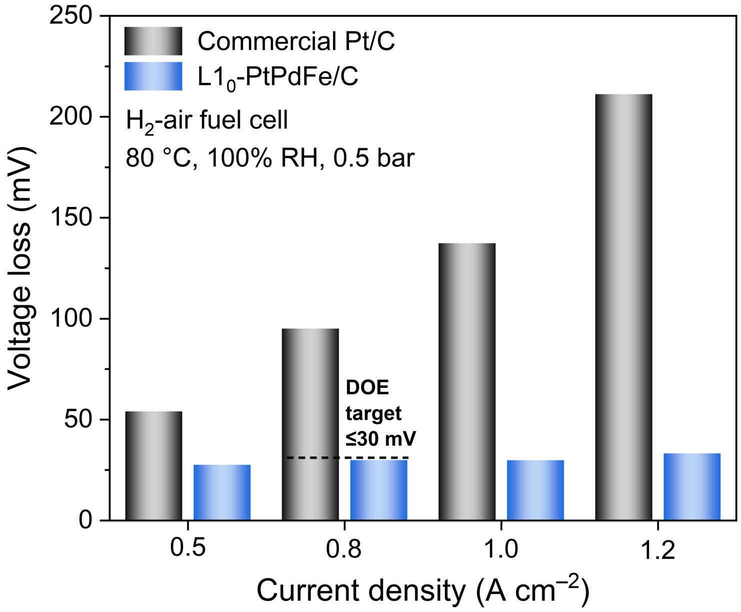

Figure S24. Voltage loss for commercial Pt/C and L1$_0$-PtPdFe/C after 30,000 ADT cycles at different current densities under H_2–air condition.

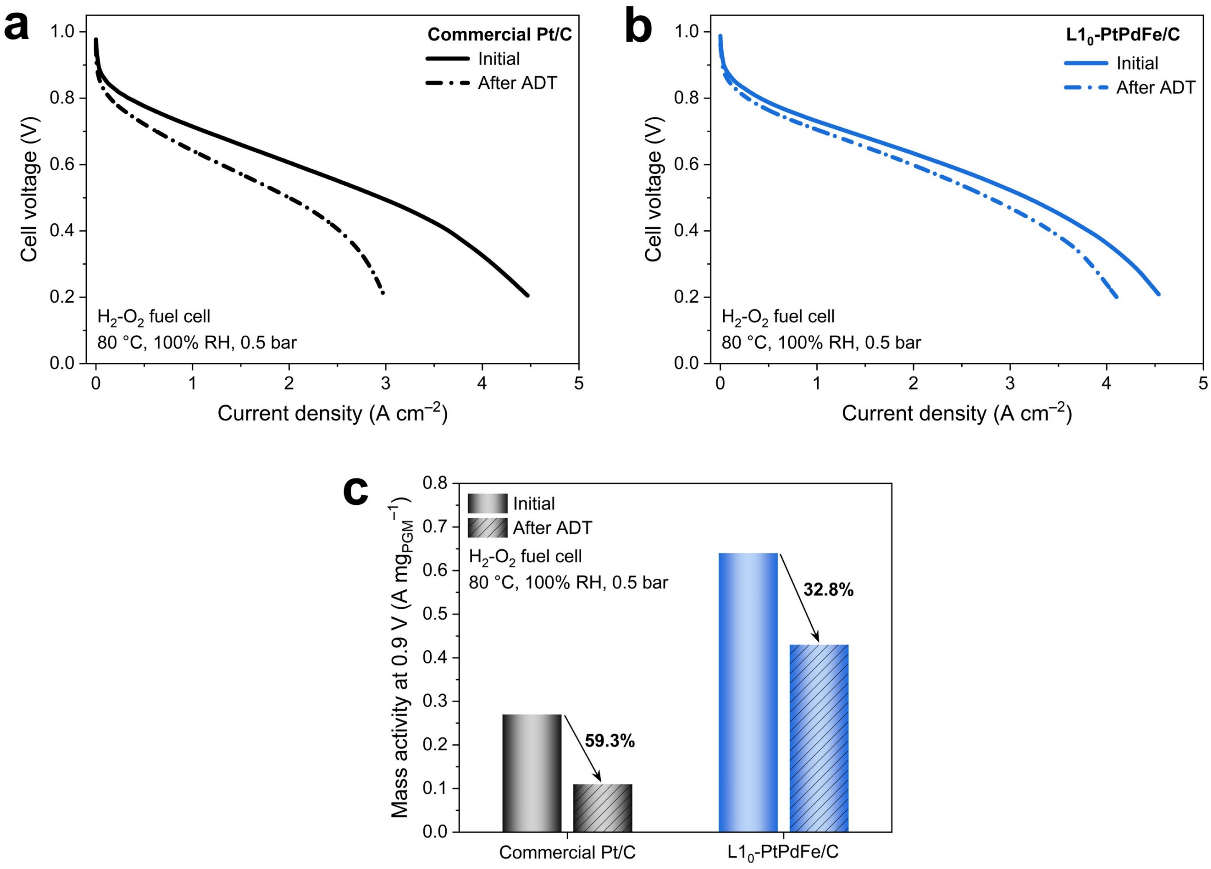

Figure S25. H_2–O_2 polarization curves of a) commercial Pt/C and b) $L1_0$-PtPdFe/C before and after 30,000 ADT cycles and c) their corresponding MA at 0.9 V.

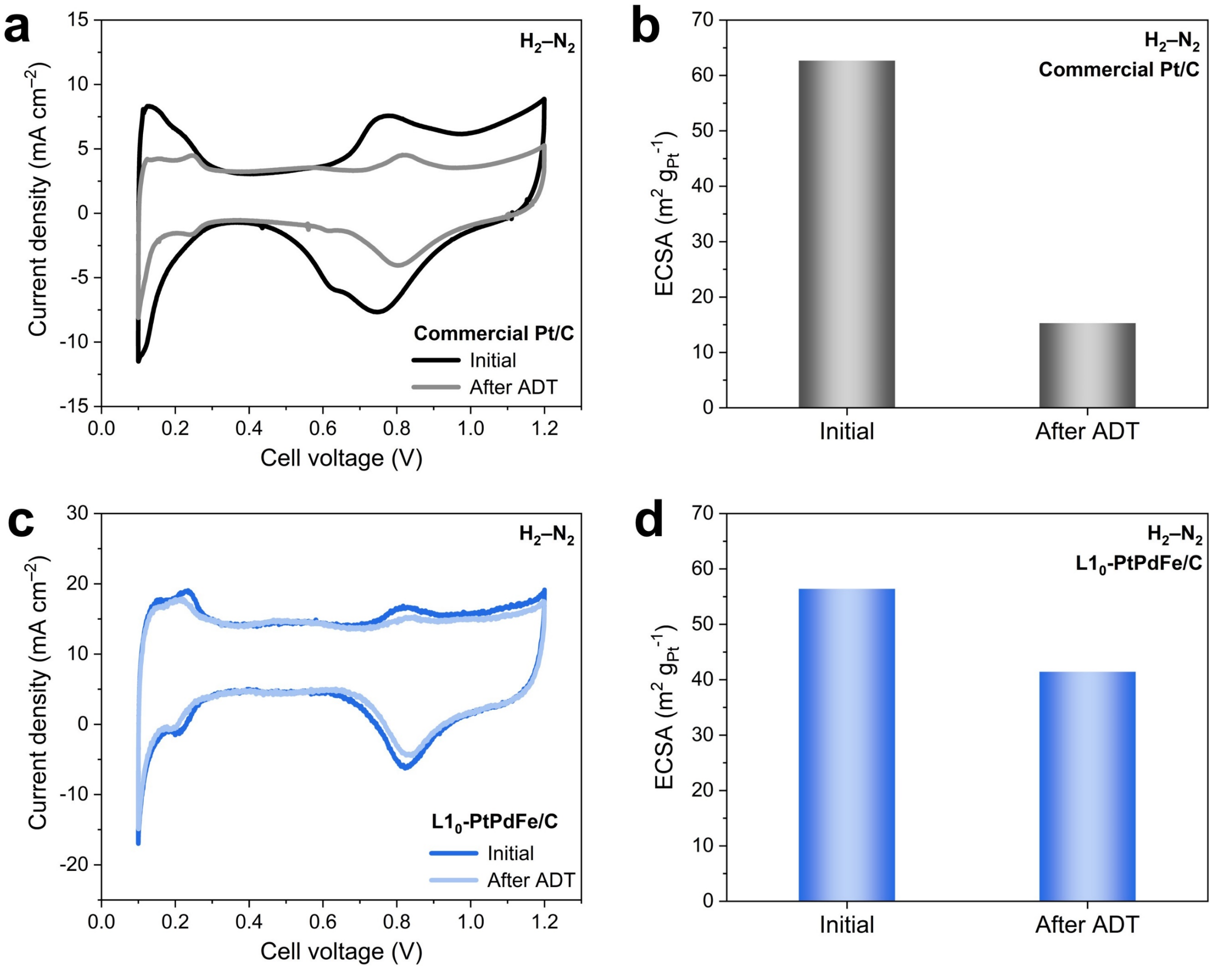

Figure S26. CV and ECSA recorded in the single-cell test in H_2–N_2 for a,b) commercial Pt/C and c,d) $L1_0$-PtPdFe/C, respectively, before and after 30,000 cycles of ADT.

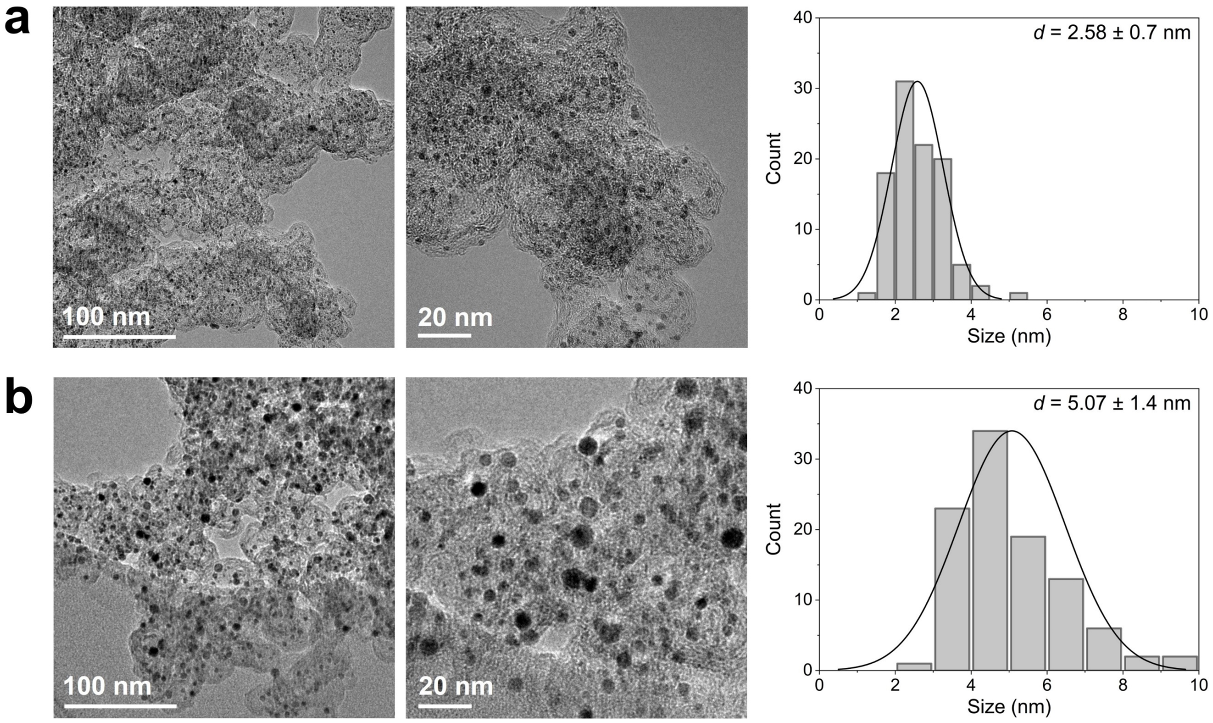

Figure S27. TEM images and their particle size distribution histograms of commercial Pt/C a) before and b) after ADT.

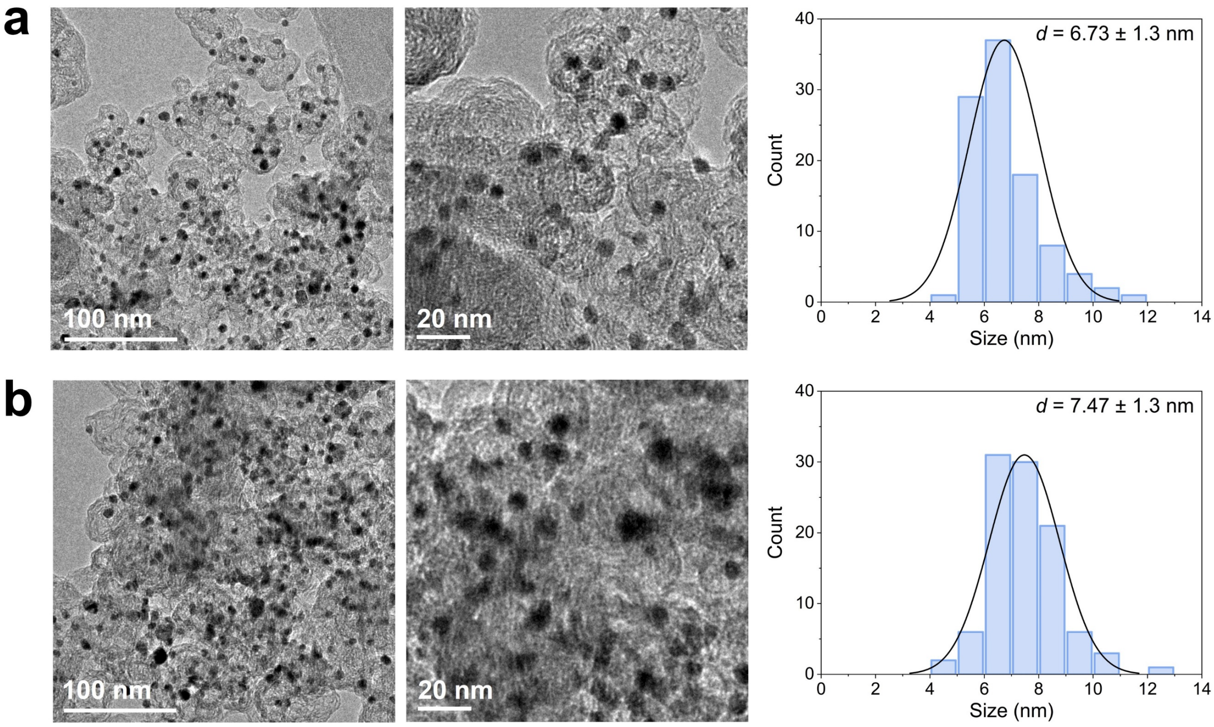

Figure S28. TEM images and their particle size distribution histograms of L1_0-PtPdFe/C a) before and b) after ADT.

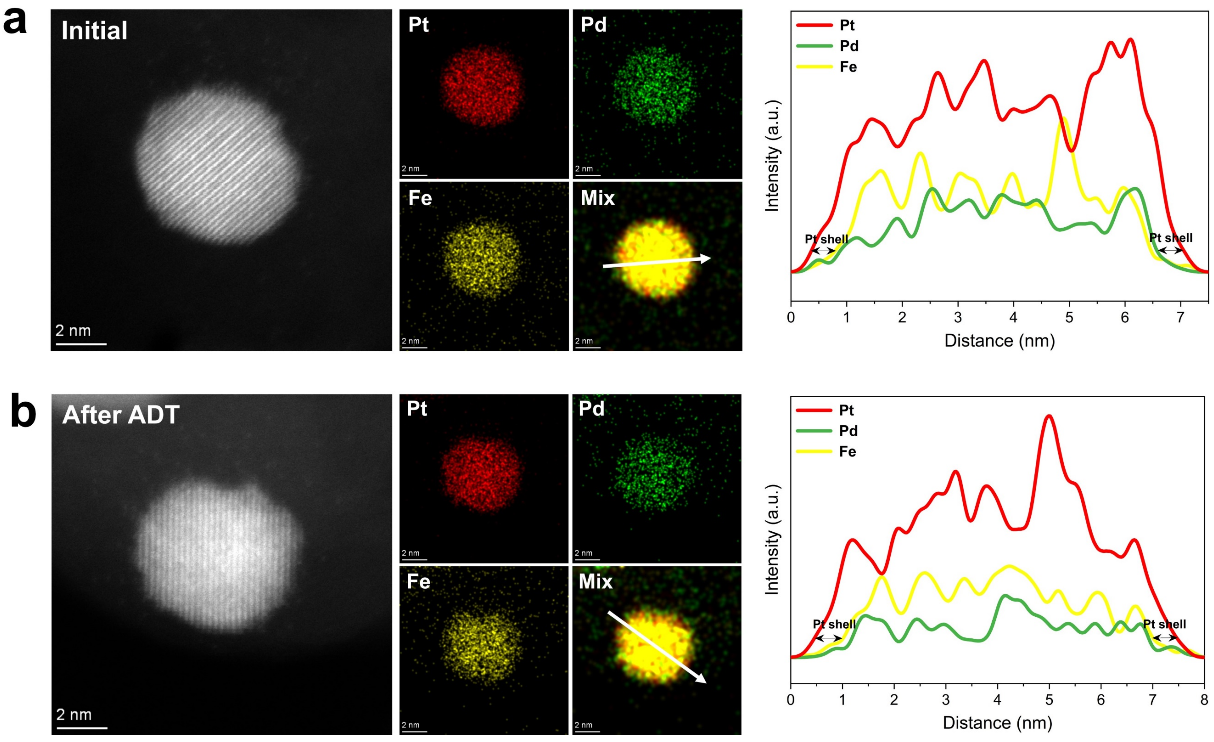

Figure S29. STEM-EDS elemental mapping and line intensity profiles of $L1_0$-PtPdFe/C MICs a) before and b) after 30,000 ADT cycles.

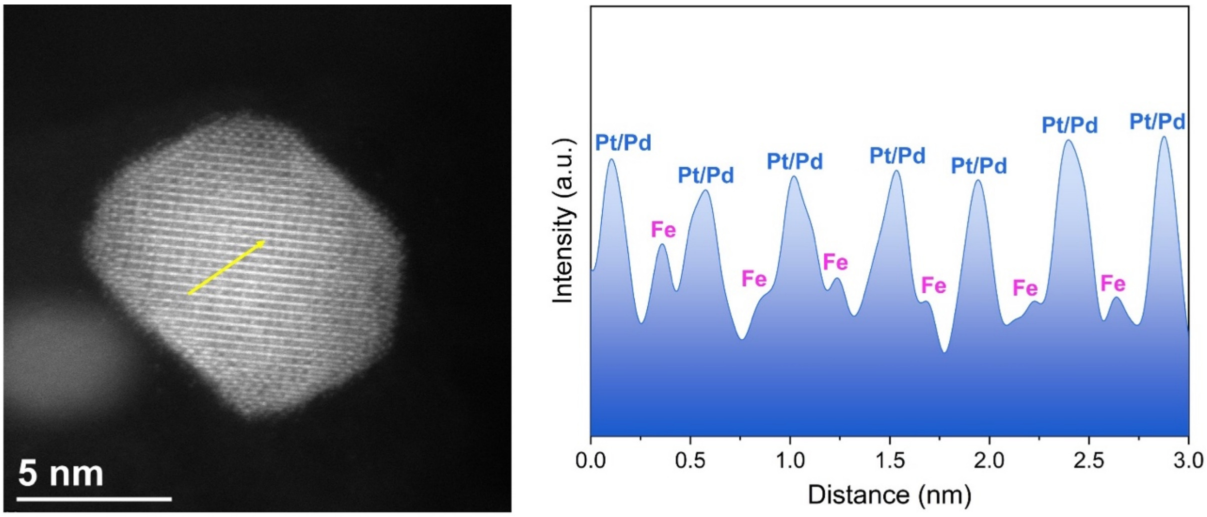

Figure S30. Atomic-resolution HAADF-STEM image of $L1_0$-PtPdFe/C particle and its corresponding scanning line profile after 30,000 ADT cycles.

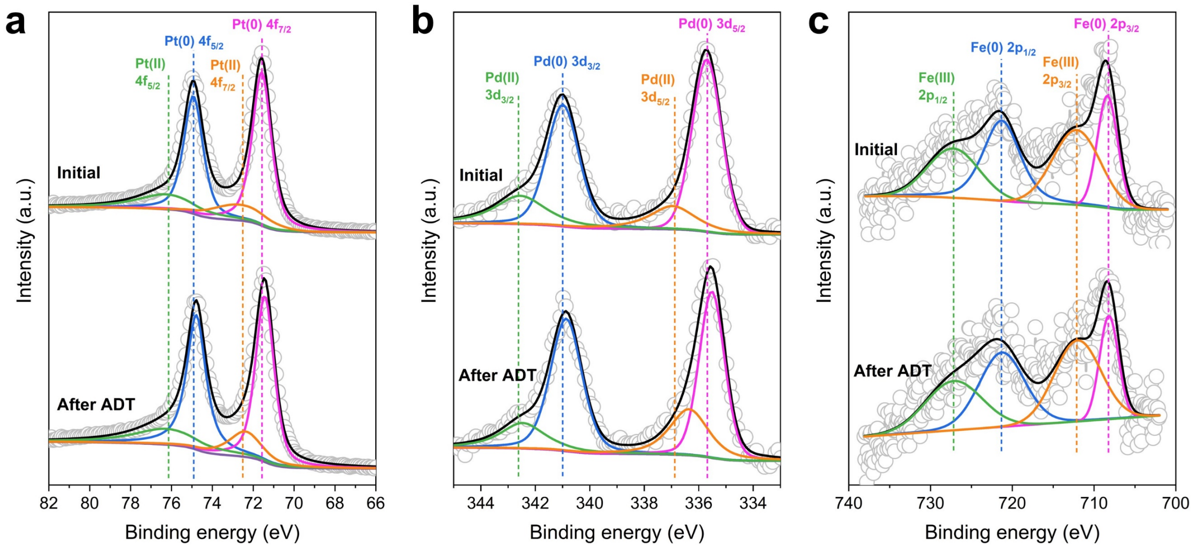

Figure S31. High-resolution a) Pt 4f, b) Pd 3d, and c) Fe 2p XPS spectra of $L1_0$-PtPdFe/C MICs at initial and after 30,000 ADT cycles.

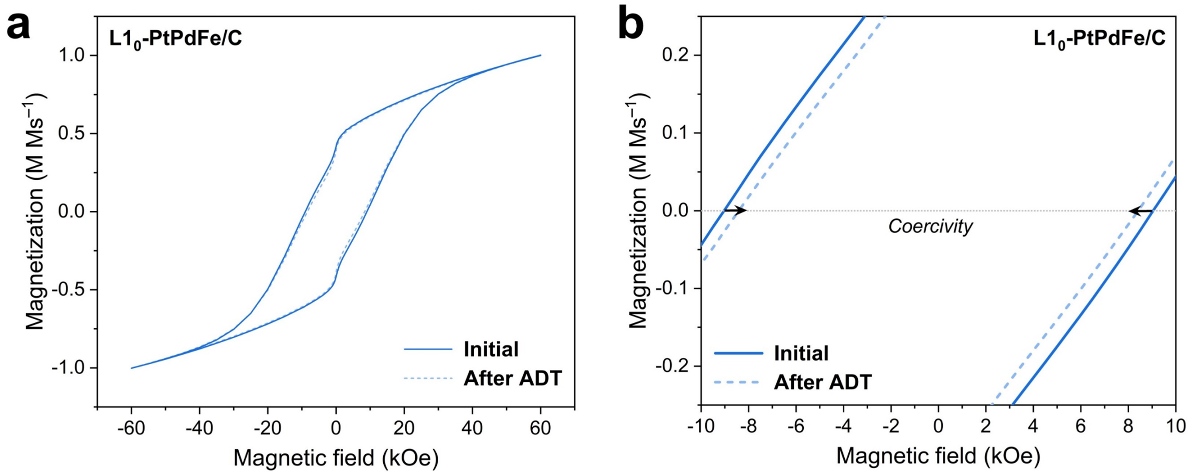

Figure S32. a) Full magnetic hysteresis (M–H) loops and b) enlarged view of the M–H curves measured at room temperature along out-of-plane direction for the CCM of $L1_0$-PtPdFe/C cathode catalysts at initial and after ADT in MEA single-cell configuration.

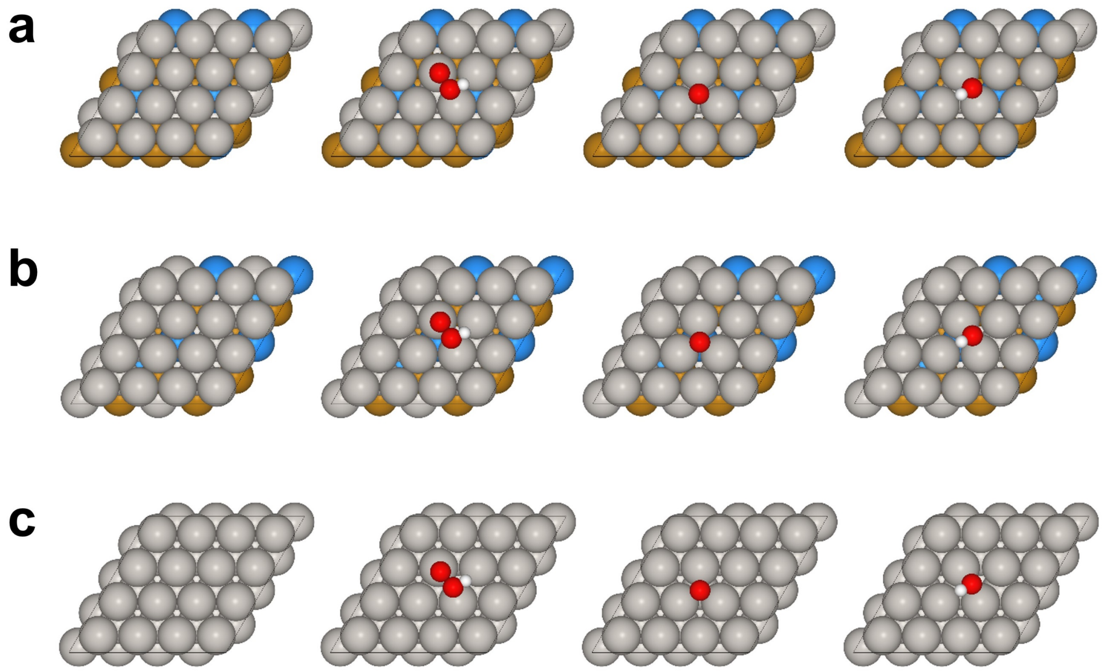

Figure S33. Top view of optimized intermediates structures on a) $L1_0$-PtPdFe alloy, b) $L1_2$-PtPdFe alloy, and c) Pt, in the order of bare surface, OOH*, O*, and OH*.

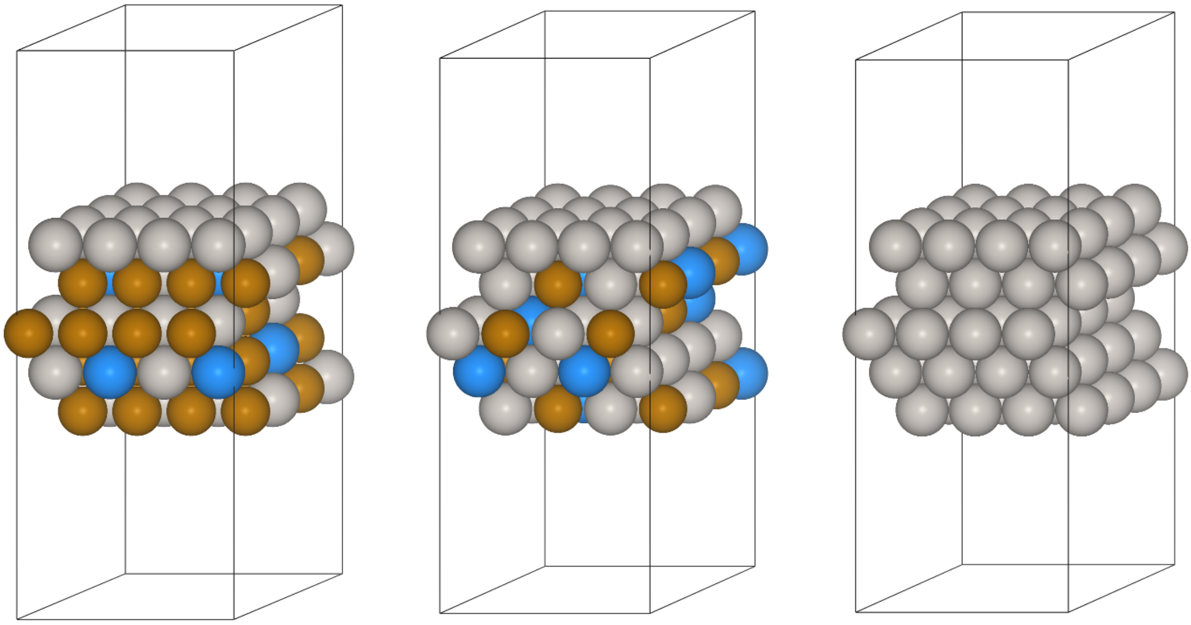

Figure S34. Constructed (111) surface images of $L1_0$-PtPdFe alloy (left), $L1_2$-PtPdFe alloy (middle), and Pt (right).

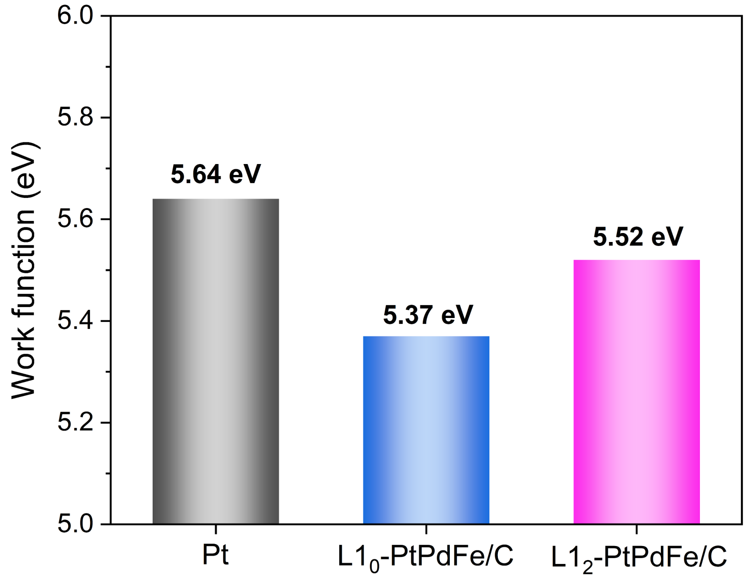

Figure S35. The calculated work function of each system.

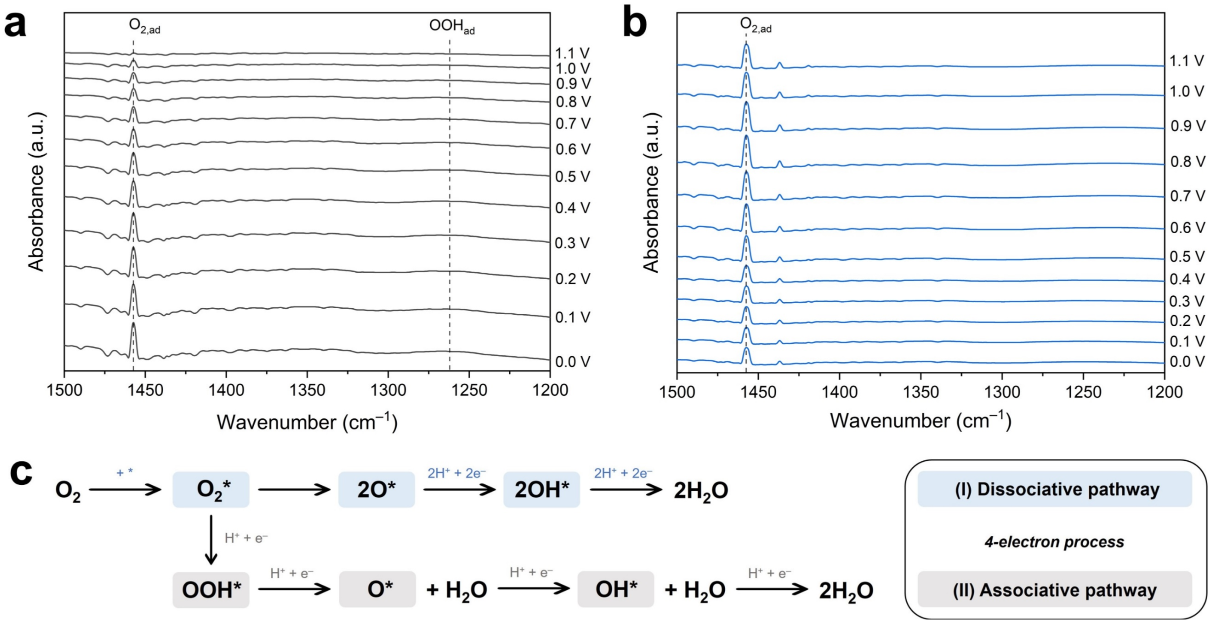

Figure S36. In situ ATR-FTIR spectra under applied potentials during ORR in O_2-saturated 0.1 M $HClO_4$ electrolyte for a) commercial Pt/C and b) $L1_0$-PtPdFe/C MICs. c) Schematic illustration of the proposed ORR mechanism on a catalyst surface in acidic media.

Table S1. Structural parameters of the $L1_0$-PtPdFe/C and $L1_2$-PtPdFe/C MICs from the Pt L_3-edge EXAFS fittings.

Sample	Path	CN*	d* (Å)	σ^2* (Å^2)	R-factor*
$L1_0$-PtPdFe/C	Pt–Pt/Pd	6.5 ± 1.7	2.693 ± 0.008	0.0067 ± 0.0013	0.018
	Pt–Fe	3.6 ± 1.0	2.655 ± 0.013	0.0086 ± 0.0023	
$L1_2$-PtPdFe/C	Pt–Pt/Pd	7.8 ± 2.0	2.702 ± 0.007	0.0077 ± 0.0012	0.026
	Pt–Fe	3.4 ± 1.8	2.673 ± 0.024	0.0124 ± 0.0052	

*CN, coordination numbers; d, distance between absorber and backscatter atoms; σ^2, Debye-Waller factor; R-factor, a measure of the quality of the EXAFS fit.

Table S2. XRD crystallite size and ordering degree of the $L1_0$-PtPdFe/C transformed from A1-PtPdFe-11/C NPs thermally treated at different annealing temperatures.

Sample	Temperature, holding time	Crystallite size* (nm)	Ordering degree** (%)
$L1_0$-PtPdFe-400/C	400 °C, 6 h	2.49	-
$L1_0$-PtPdFe-500/C	500 °C, 6 h	2.95	-
$L1_0$-PtPdFe-600/C	600 °C, 6 h	3.31	23
$L1_0$-PtPdFe-700/C	700 °C, 6 h	4.78	32
$L1_0$-PtPdFe-800/C	800 °C, 6 h	6.03	69
$L1_0$-PtPdFe-900/C	900 °C, 6 h	8.06	52
$L1_0$-PtPdFe-1000/C	1000 °C, 6 h	8.66	35

*Crystallite sizes were calculated using Debye–Scherrer equation.

**Ordering degrees were calculated using the ratio of the integrated area under (110) signal to the sum of the areas under (111), (200), and (002) signals, $S_{(110)}/(S_{(111)} + S_{(200)} + S_{(002)})$. The standard value of $S_{(110)}/(S_{(111)} + S_{(200)} + S_{(002)})$ for $L1_0$-PtFe (PDF #03-065-9121) is 0.188, which corresponds to 100% ordering degree.

Table S3. XRD crystallite size and ordering degree of the $L1_2$-PtPdFe/C transformed from A1-PtPdFe-31/C NPs thermally treated at different annealing temperatures.

Sample	Temperature, holding time	Crystallite size* (nm)	Ordering degree** (%)
$L1_2$-PtPdFe-400/C	400 °C, 6 h	3.33	-
$L1_2$-PtPdFe-500/C	500 °C, 6 h	3.99	-
$L1_2$-PtPdFe-600/C	600 °C, 6 h	4.47	-
$L1_2$-PtPdFe-700/C	700 °C, 6 h	5.11	17
$L1_2$-PtPdFe-800/C	800 °C, 6 h	6.54	40
$L1_2$-PtPdFe-900/C	900 °C, 6 h	7.01	67
$L1_2$-PtPdFe-1000/C	1000 °C, 6 h	9.22	57

*Crystallite sizes were calculated using Debye–Scherrer equation.

**Ordering degrees were calculated using the ratio of the integrated area under (110) signal to the sum of the areas under (111) and (200) signals, $S_{(110)}/(S_{(111)} + S_{(200)})$. The standard value of $S_{(110)}/(S_{(111)} + S_{(200)})$ for $L1_2$-PtFe (PDF #01-089-2050) is 0.085, which corresponds to 100% ordering degree.

Table S4. Magnetocrystalline anisotropy energy and key magnetic parameters for $L1_0$-PtPdFe/C and $L1_2$-PtPdFe/C MICs.

**Sample**	$\boldsymbol{K_u}$* (J m^{-3})	$\boldsymbol{H_c}$** (kOe)	$\boldsymbol{M_s}$*** (emu g^{-1})
$L1_0$-PtPdFe/C	13.41×10^4	23.8	7.95
$L1_2$-PtPdFe/C	7.58×10^4	10.1	8.93

*The effective magnetocrystalline anisotropy energy density K_u was calculated using the equation:

$$K_u = \frac{1}{2}\mu_0 H_c M_s$$

where μ_0 is the vacuum permeability ($\mu_0 = 4\pi \times 10^{-7}$ T m A^{-1}), H_c is the magnetic coercivity field (converted to A m^{-1}), and M_s is the saturation magnetization (converted to A m^{-1}).
**The coercivity H_c in kOe was converted to A m^{-1} using the relation 1 Oe = 79.577 A m^{-1}.
***The saturation magnetization M_s in emu g^{-1} was converted to A m^{-1} using the relation 1 emu cm^{-3} = 1000 A m^{-1} and respective alloy density ρ (g cm^{-3}) (see Tables S5 and S6, Supporting Information).

Table S5. Calculation of alloy density for $L1_0$-PtPdFe (total atoms = 2.0).

**Element**	**Amount** (mol)	***M*** (g mol^{-1})	***ρ*** (g cm^{-3})	**M × mol**	**(M/*ρ*) × mol**
Pt	0.75	195.08	21.45	146.31	6.82
Pd	0.25	106.42	12.02	26.61	2.21
Fe	1.00	55.85	7.87	55.85	7.10
**Total**				228.77	16.13
**Calculated alloy density*:** ρ **$L1_0$-PtPdFe** $= \frac{228.77}{16.13} \approx$ **14.18 g cm^{-3}**					

*Alloy density was calculated using the rule of mixtures (mass-based) for metallic alloys:

$$\rho_{\text{alloy}} = \frac{\sum x_i M_i}{\sum x_i \frac{M_i}{\rho_i}}$$

where x_i is the atomic ratio, M_i is the molar mass (g mol^{-1}), and ρ_i is the elemental density (g cm^{-3}) of element i.

Table S6. Calculation of alloy density for $L1_2$-PtPdFe (total atoms = 4.0).

Element	**Amount** (mol)	***M*** (g mol^{-1})	***ρ*** (g cm^{-3})	**M × mol**	**(M/*ρ*) × mol**
Pt	2.25	195.08	21.45	438.93	20.46
Pd	0.75	106.42	12.02	79.82	6.64
Fe	1.00	55.85	7.87	55.85	7.10
**Total**				574.60	34.20
**Calculated alloy density*:** $\rho\ L1_2\text{-PtPdFe} = \frac{574.60}{34.20} \approx 16.80\ \text{g cm}^{-3}$					

*Alloy density was calculated using the rule of mixtures (mass-based) for metallic alloys:

$$\rho_{\text{alloy}} = \frac{\sum x_i M_i}{\sum x_i \frac{M_i}{\rho_i}}$$

where x_i is the atomic ratio, M_i is the molar mass (g mol^{-1}), and ρ_i is the elemental density (g cm^{-3}) of element i.

Table S7. Calculated spin magnetic moments (μ_s), orbital magnetic moments (μ_L), and total magnetic moments (μ_J) of bulk $L1_0$-PtPdFe and $L1_2$-PtPdFe crystal under [100] and [001] magnetization directions.

**Unit: μ_B/atom**		**$L1_0$-PtPdFe**			**$L1_2$-PtPdFe**		
		**Pt**	**Pd**	**Fe**	**Pt**	**Pd**	**Fe**
[100]	μ_s	0.351	0.320	2.989	0.350	0.313	3.135
	μ_L	0.076	0.018	0.069	0.066	0.008	0.080
	μ_J	0.427	0.338	3.058	0.416	0.321	3.215
[001]	μ_s	0.352	0.320	2.989	0.348	0.313	3.135
	μ_L	0.071	0.001	0.071	0.079	0.009	0.085
	μ_J	0.423	0.321	3.060	0.427	0.322	3.220

Table S8. Lattice constant and strain of the prepared electrocatalysts based on the XRD results.

**Sample**	**Lattice constant** (Å)		**Strain*** (%)		**Initial MA** (A mg_{PGM}^{-1})
	**a, b**	**c**	**a, b**	**c**	
Pt (#01-087-0636)	3.9440	3.9440	-	-	-
A1-PtPdFe-11/C	3.8522	3.8522	2.33	2.33	0.28
A1-PtPdFe-31/C	3.8960	3.8960	1.22	1.22	0.20
$L1_0$-PtPdFe/C	3.8369	3.7372	2.71	5.24	0.89
$L1_2$-PtPdFe/C	3.8639	3.8639	2.03	2.03	0.56
$L1_0$-PtFe/C	3.8069	3.7070	3.48	6.01	0.95
$L1_2$-PtFe/C	3.8616	3.8616	2.09	2.09	0.66

*The compressive strain was determined using the equation $\varepsilon = (a_{Pt} - a)/a_{Pt}$, where a_{Pt} is the lattice constant of pure Pt and a represents the measured lattice constant a, b, and c of the alloy in a unit cell.[14,15]

Table S9. Elemental composition of the as-prepared electrocatalysts determined by ICP-OES.

Sample	Metal loadings			Atomic ratio		
	Pt (wt.%)	Pd (wt.%)	Fe (wt.%)	Pt (at.%)	Pd (at.%)	Fe (at.%)
$L1_0$-PtPdFe/C	16.2	2.8	4.8	42.5	13.5	44.0
$L1_2$-PtPdFe/C	19.2	3.6	2.1	57.9	19.9	22.1

Table S10. Comparative fuel cell performance of our L1_0-PtPdFe/C MICs with reported state-of-the-art Pt-based electrocatalysts under MEA test conditions.

**Catalyst**	**H_2–O_2 fuel cells**			**H_2–N_2 fuel cells**			**H_2–air fuel cells**	**Reference**
	**Mass activity at 0.9 V** (A mg_{PGM}^{-1})		**MA loss** (%)	**ECSA** (m^2 g_{Pt}^{-1})		**ECSA loss** (%)	**Voltage loss at 0.8 A cm^{-2}** (mV)	
	**Initial**	**After ADT**		**Initial**	**After ADT**			
L1_0-PtPdFe/C	0.64	0.43	32.8	56.4	41.4	26.5	29	This work
i-CoPt@Pt/KB	0.53	0.34	35.5	53.3	43.3	18.8	29	[16]
PtCo@Gnp	1.14	0.75	34.2	75.3	48.5	35.6	23	[17]
Int-PtNiN/KB	0.49	0.32	34.7	60.9	30.4	50.1	32	[18]
L1_0-Cr-PtFe/C	1.02	0.76	25.5	-	-	-	10	[19]
L1_0-CoPt@Pt-shell	0.60	0.36	40.0	52.0	34.8	33.1	26	[20]
L1_0-CoPt/Pt	0.56	0.45	19.6	26.4	23.0	12.9	69	[21]
PtFe-H/Pt	0.92	0.70	23.9	-	-	-	33	[22]
L1_0-$Pt_{50}Ni_{35}Ga_{15}$/C	0.70	0.52	25.7	-	-	-	16	[23]
Pt_3Co/FeN_4-C	0.72	0.44	38.9	-	-	-	21	[24]

Table S11. Elemental composition of the $L1_0$-PtPdFe/C electrocatalysts at initial and after 30,000 ADT cycles determined by EDS and XPS analysis.

Analysis	Initial			After ADT		
	**Pt (at.%)**	**Pd (at.%)**	**Fe (at.%)**	**Pt (at.%)**	**Pd (at.%)**	**Fe (at.%)**
EDS	45.2	14.5	40.3	58.2	11.9	29.9
XPS	43.5	13.6	42.9	56.5	11.4	32.1

References

[1] G. Kresse and J. Furthmü, "Efficient Iterative Schemes for Ab Initio Total-Energy Calculations Using a Plane-Wave Basis Set," *Physical Review B* 54 (1996): 11169–11186. https://doi.org/10.1103/PhysRevB.54.11169

[2] G. Kresse and J. Furthmüller, "Efficiency of Ab-Initio Total Energy Calculations for Metals and Semiconductors Using a Plane-Wave Basis Set," *Computational Materials Science* 6, no. 1 (1996): 15–50. https://doi.org/10.1016/0927-0256(96)00008-0

[3] P. E. Blochl, "Projector Augmented-Wave Method," *Physical Review B* 50 (1994): 17953–17979. https://doi.org/10.1103/PhysRevB.50.17953

[4] G. Kresse and D. Joubert, "From Ultrasoft Pseudopotentials to the Projector Augmented-Wave Method," *Physical Review B* 59 (1999): 1758–1775. https://doi.org/10.1103/PhysRevB.59.1758

[5] J. P. Perdew, K. Burke, and M. Ernzerhof, "Generalized Gradient Approximation Made Simple," *Physical Review Letters* 77 (1996): 3865–3868. https://doi.org/10.1103/PhysRevLett.77.3865

[6] B. Błoński and J. Hafner, "Density-Functional Theory of the Magnetic Anisotropy of Nanostructures: An Assessment of Different Approximations," *Journal of Physics: Condensed Matter* 21, no. 42 (2009): 426001. https://doi.org/10.1088/0953-8984/21/42/426001

[7] S. Grimme, "Semiempirical GGA-Type Density Functional Constructed with a Long-Range Dispersion Correction," *Journal of Computational Chemistry* 27, no. 15 (2006): 1787–1799. https://doi.org/10.1002/jcc.20495

[8] S. Grimme, J. Antony, S. Ehrlich, and H. Krieg, "A Consistent and Accurate *Ab Initio* Parametrization of Density Functional Dispersion Correction (*DFT-D*) for the 94 Elements H–Pu," *The Journal of Chemical Physics* 132 (2010): 154104. https://doi.org/10.1063/1.3382344

[9] H. J. Monkhorst and J. D. Pack, "Special Points for Brillouin-Zone Integrations," *Physical Review B* 13 (1976): 5188–5192. https://doi.org/10.1103/PhysRevB.13.5188

[10] B. J. Morgan, "bsym: A Basic Symmetry Module," *Journal of Open Source Software* 2, no. 16 (2017): 370. https://doi.org/10.21105/joss.00370

[11] T. Xie and J. C. Grossman, "Crystal Graph Convolutional Neural Networks for an Accurate and Interpretable Prediction of Material Properties," *Physical Review Letters* 120 (2018): 145301. https://doi.org/10.1103/PhysRevLett.120.145301

[12] J. K. Nørskov, J. Rossmeisl, A. Logadottir, et al., "Origin of the Overpotential for Oxygen Reduction at a Fuel-Cell Cathode," *The Journal of Physical Chemistry B* 108, no. 46 (2004): 17886–17892. https://doi.org/10.1021/jp047349j

[13] V. Wang, N. Xu, J. C. Liu, G. Tang, and W. T. Geng, "VASPKIT: A User-Friendly Interface Facilitating High-Throughput Computing and Analysis Using VASP Code," *Computer Physics Communications* 267 (2021): 108033. https://doi.org/10.1016/j.cpc.2021.108033

[14] Z. Xing, J. Li, S. Wang, C. Su, and H. Jin, "Structure Engineering of $PtCu_3/C$ Catalyst from Disordered to Ordered Intermetallic Compound with Heat-Treatment for the Methanol Electrooxidation Reaction," *Nano Research* 15 (2022): 3866–3871. https://doi.org/10.1007/s12274-021-3993-8

[15] M. I. Maulana, H.-Y. Lee, C. Gyan-Barimah, J. H. Sung, and J.-S. Yu, "Hollow PtCo Alloy Nanostructures for Efficient Oxygen Reduction Electrocatalysis in Polymer Electrolyte Membrane Fuel Cells," *Journal of Materials Chemistry A* 12 (2024): 27979–27986. https://doi.org/10.1039/D4TA04470C

[16] T. Y. Yoo, J. Lee, S. Kim, et al., "Scalable Production of an Intermetallic Pt–Co Electrocatalyst for High-Power Proton-Exchange-Membrane Fuel Cells," *Energy & Environmental Science* 16 (2023): 1146–1154. https://doi.org/10.1039/D2EE04211H

[17] Z. Zhao, Z. Liu, A. Zhang, et al., "Graphene-Nanopocket-Encaged PtCo Nanocatalysts for Highly Durable Fuel Cell Operation under Demanding Ultralow-Pt-Loading Conditions," *Nature Nanotechnology* 17 (2022): 968–975. https://doi.org/10.1038/s41565-022-01170-9

[18] X. Zhao, C. Xi, R. Zhang, Let al., "High-Performance Nitrogen-Doped Intermetallic PtNi Catalyst for the Oxygen Reduction Reaction," *ACS Catalysis* 10 (18): 10637–10645. https://doi.org/10.1021/acscatal.0c03036

[19] X. Liu, Y. Wang, J. Liang, et al., "Introducing Electron Buffers into Intermetallic Pt Alloys against Surface Polarization for High-Performing Fuel Cells," *Journal of the American Chemical Society* 146 (3): 2033–2042. https://doi.org/10.1021/jacs.3c10681

[20] Y. T. Pan, D. Li, S. Sharma, et al., "Ordered CoPt Oxygen Reduction Catalyst with High Performance and Durability," *Chem Catalysis* 2, no. 12 (2022): 3559–3572. https://doi.org/10.1016/j.checat.2022.10.030

[21] J. Li, S. Sharma, X. Liu, et al., "Hard-Magnet $L1_0$-CoPt Nanoparticles Advance Fuel Cell Catalysis," *Joule* 3, no. 1 (2019): 124–135. https://doi.org/10.1016/j.joule.2018.09.016

[22] T. Song, M. Chen, P. Yin, et al., Small 2022, "Intermetallic PtFe Electrocatalysts for the Oxygen Reduction Reaction: Ordering Degree-Dependent Performance," *Small* 18, no. 31 (2022): 2202916. https://doi.org/10.1002/smll.202202916

[23] J. Liang, Y. Wan, H. Lv, et al., "Metal Bond Strength Regulation Enables Large-Scale Synthesis of Intermetallic Nanocrystals for Practical Fuel Cells," *Nature Materials* 23 (2024): 1259–1267. https://doi.org/10.1038/s41563-024-01901-4

[24] Z. Qiao, C. Wang, C. Li, et al., "Atomically Dispersed Single Iron Sites for Promoting Pt and Pt_3Co Fuel Cell Catalysts: Performance and Durability Improvements," *Energy & Environmental Science* 14 (2021): 4948–4960. https://doi.org/10.1039/D1EE01675J
